# Integrated network analysis and logistic regression modeling identify stage-specific genes in Oral Squamous Cell Carcinoma

**DOI:** 10.1186/s12920-015-0114-0

**Published:** 2015-07-16

**Authors:** Vinay Randhawa, Vishal Acharya

**Affiliations:** 1 grid.417640.0000000040500553XFunctional Genomics and Complex Systems Laboratory, Biotechnology Division, CSIR-Institute of Himalayan Bioresource Technology, Council of Scientific and Industrial Research, Palampur, Himachal Pradesh India; 2 Academy of Scientific and Innovative Research (AcSIR), New Delhi, India

**Keywords:** Coexpression network analysis, Gene module, Hub gene, Microarray, Oral squamous cell carcinoma, Logistic regression modeling

## Abstract

**Background:**

Oral squamous cell carcinoma (OSCC) is associated with substantial mortality and morbidity but, OSCC can be difficult to detect at its earliest stage due to its molecular complexity and clinical behavior. Therefore, identification of key gene signatures at an early stage will be highly helpful.

**Methods:**

The aim of this study was to identify key genes associated with progression of OSCC stages. Gene expression profiles were classified into cancer stage-related modules, i.e., groups of genes that are significantly related to a clinical stage. For prioritizing the candidate genes, analysis was further restricted to genes with high connectivity and a significant association with a stage. To assess predictive power of these genes, a classification model was also developed and tested by 5-fold cross validation and on an independent dataset.

**Results:**

The identified genes were enriched for significant processes and functional pathways, and various genes were found to be directly implicated in OSCC. Forward and stepwise, multivariate logistic regression analyses identified 13 key genes whose expression discriminated early- and late-stage OSCC with predictive accuracy (area under curve; AUC) of ~0.81 in a 5-fold cross-validation strategy.

**Conclusions:**

The proposed network-driven integrative analytical approach can identify multiple genes significantly related to an OSCC stage; the classification model that is developed with these genes may help to distinguish cancer stages. The proposed genes and model hold promise for monitoring of OSCC stage progression, and our findings may facilitate cancer detection at an earlier stage, resulting in improved treatment outcomes.

**Electronic supplementary material:**

The online version of this article (doi:10.1186/s12920-015-0114-0) contains supplementary material, which is available to authorized users.

## Background

Oral cancer is a common cancer worldwide with squamous cell carcinoma being the most prevalent subtype, which accounts for 96 % of oral-cavity cancers [1]. There are ~260,000 new cases of oral squamous cell carcinoma (OSCC) and 124,000 deaths worldwide annually [2]. Despite considerable advances in treatments, the overall five-year survival rate of patients at the advanced stage is only 30 %, but is greater than 90 % among patients with early-stage OSCC [3]. Unfortunately, only 35 % of cases of oral cancer are detected at the earliest stage (without producing symptoms) [4]because of its molecular complexity and clinical behavior. Therefore, there is an urgent need for identifying molecular predictors that may enable cancer detection at early stages.

Remarkable advances in technologies for assaying gene expression and the availability of high-throughput data have opened up new avenues of cancer research that may allow researchers to generate hypotheses regarding improved disease classification. Global gene expression profiles in OSCC have been studied using traditional approaches which have helped to identify some candidate gene biomarkers on the basis of comparison between cancerous and non-cancerous cases [5–7]. In addition, several computational methods have been developed to identify biomarkers for oral cancer prognosis and diagnosis [8–10]. Comparative analysis of expression profiles between early and late stages has uncovered genes with stage-dependent alterations in expression of various cancers [11, 12]; however, to our knowledge, not much efforts has been devoted to analysis of OSCC stage progression in relation to aggressiveness of the cancer.

Genes and proteins function cooperatively and thus regulate common biological processes by co-regulating each other [13]; however, genes identified via the classical approaches are usually not functionally related and therefore may not reveal key biological processes. Because of these limitations, the traditional approaches are not very useful for identification of specific genes that contribute to or are affected by complex diseases. Fortunately, rapid advances in network biology have effectively provided valuable frameworks for analysis of multidimensional biological data and have important applications to clinical practice. Instead of analyzing tens of thousands of gene comparisons, the network-based analysis offers a meaningful data reduction scheme that limits the analysis to only hundreds [14–16] or even tens [17–20] of relevant genes. Altered gene coexpression networks have been proven to be the major cause of dysregulated expression during cancer progression [21, 22]. Diseases can therefore be studied as networks by systematically exploring topological associations between contributing genes. Gene coexpression networks have been utilized even to identify key tumorigenic genes with the aim to find biomarkers or to gain insights into probable disease mechanisms. Nevertheless, most of these studies remain limited to physically interacting genes and do not take into account their associations with the disease phenotype. On the other hand, disruption in connections within disease modules give rise to particular disease phenotypes [23]. Thus, now it seems to be more important to consider the phenotypic association in order to characterize the mechanisms of disease progression [24].

A complex alteration of global gene expression profiles among genes is a determinant of progression of cancer stages and grades [25–27].Because genes that are highly connected within a gene set are thought to drive other genes [28], it was hypothesized that examining the altered gene expression profiles of key genes might help to discriminate between early and late stage cancers. Although cancer stage is an effective prognostic factor, to the best of our knowledge, systematic studies characterizing gene expression data in relation to OSCC stage progression are scarce.

In the present study, we identified key genes associated with OSCC stage and developed a classification model to discriminate the two most common OSCC groups: early stages (I, II) versus late stages (III, IV). For this purpose, first, a set of highly correlating genes was obtained from a gene coexpression network. For prioritizing the candidate genes, the analysis were then restricted to genes with high connectivity and a significant association with cancer stage. To advance the understanding of these relations, using expression profiles of the putative gene candidates, we then develop a classification model to discriminate cancer stages; this model helped to classify OSCC efficiently. The methodology presented herein seems to be the first implementation of key hub genes (depending on topological and phenotypic importance) to identify an OSCC stage and may predict clinical aggressiveness of this cancer.

## Methods

**Fig. 1:**
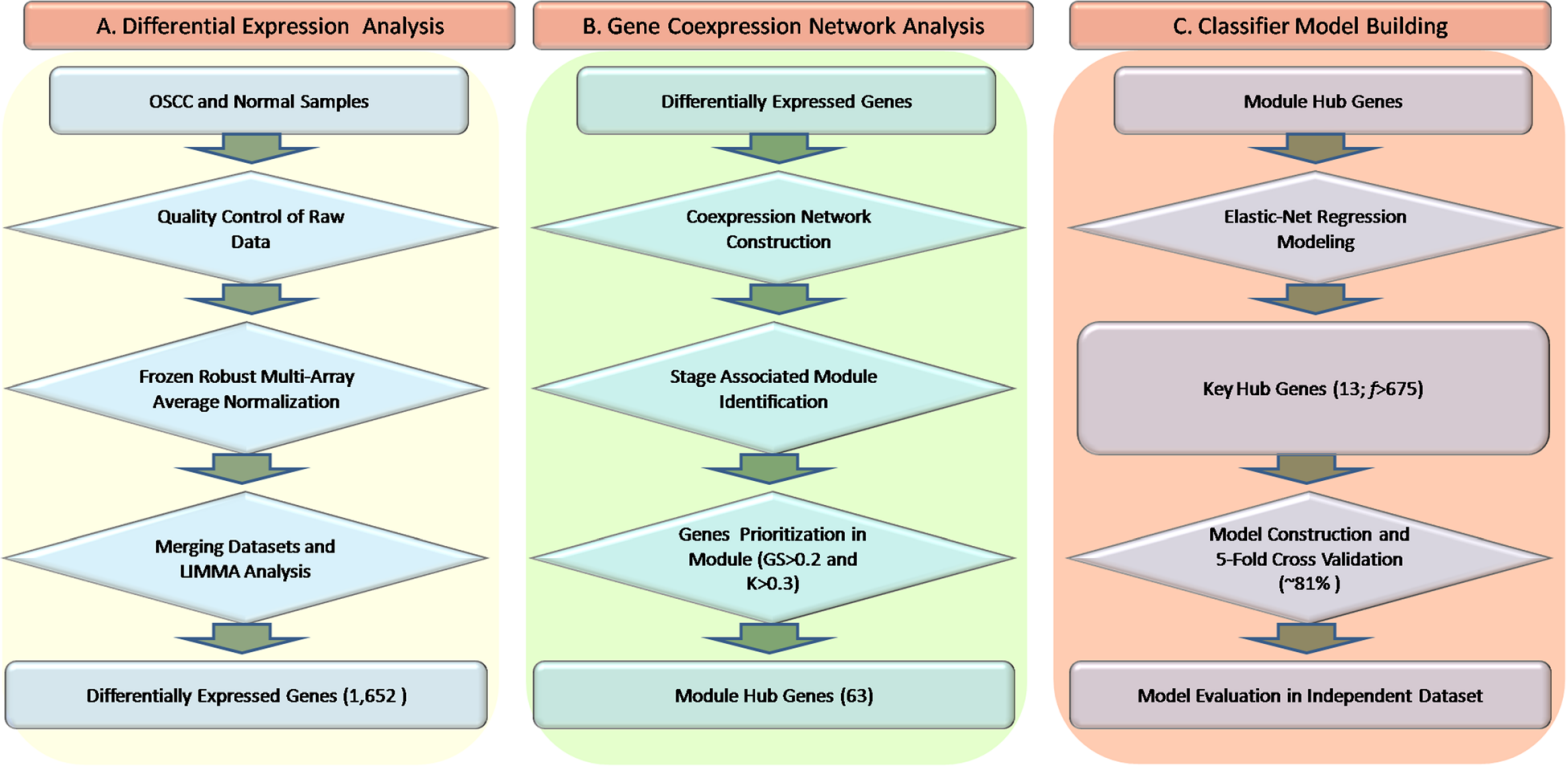
The steps involved in systems level analysis of data on oral squamous cell carcinoma (OSCC). **a** Microarray data collection and preprocessing of experiments to identify differentially expressed genes (DEGs). **b** Construction of the OSCC network and identification of an OSCC stage-associated module and of cancer hub genes. **c** Development and testing of a key hub gene-based classifier model by 5-fold cross-validation

To identify the key genes whose expression may discriminate between early- and late-stage OSCC samples, we adopted the following major steps: (1) merging of multiple microarray datasets to identify differentially expressed genes (DEGs) in tumor samples compared to normal (healthy) controls, (2) analysis of the gene coexpression network to identify stage associated modules and their key hub genes, and (3) development of a hub gene-based classifier model to distinguish OSCC stages. Schematic representation of the overall strategy is shown in Fig. 1.

### Acquisition and proecessing of gene expression data

Gene expression profiles of OSCC were obtained from the National Center for Biotechnology Information (NCBI) Gene Expression Omnibus (GEO) database [29] via queries with the search terms “*oral squamous cell carcinoma*” and “*head and neck squamous cell carcinoma*” (August, 2014) to specifically retrieve most widely used Affymetrix HGU-133a and HGU-133plus2 array datasets (Table 1). Studies that had well-defined phenotypic description of cancer stage were preferred. Other criteria included: (i) samples comprising only human tissue (not derived from cell lines) and without any history of specific treatment, (ii) studies comprising both case samples and healthy control samples (to identify disease-specific signals), and (iii) studies that were conducted on similar platforms (we wanted to obtain a high proportion of overlapping genes). Individual datasets were imported into the R 3.0.2 statistical environment (www.r-project.org) by means of the GEOquery tool of the Bioconductor software package (version 2.22.0) [30] and were processed using affy package. At initial levels of quality control (QC), all samples were pre-processed together using the standard affyQCReport and affyPLM quality assessment software packages. The Harshlight package [31] was utilized to remove the effects of spatial blemishes. As a general principle, chips with extensive defects were eliminated from the datasets. Background correction, quantile normalization and summarization were performed by means of the frozen robust multi-array average (fRMA) algorithm [32] using the frma package. Probesets were mapped to the human genome in the annotate and hgu133plus2.db software packages.

**Table 1:** A list of Affymetrix datasets used in the study

Dataset identifier	Initial number of samples (Tumor + Normal)	Samples (Tumor + Normal) left after initial preprocessing	Affymetrix platform	Reference
GSE31056	47 (23 + 24)	47 (23 + 24)	HGU-133plus2	[137]
GSE9844	38 (26 + 12)	38 (26 + 12)	HGU-133plus2	[5]
GSE30784	212 (167 + 45)	210 (165 + 45)	HGU-133plus2	[138]
GSE3524	20 (16 + 4)	20 (16 + 4)	HGU-133a	[139]
GSE42743	103 (74 + 29)	100 (73 + 27)	HGU-133plus2	[96]
GSE2280	27 (22 + 5)	27 (22 + 5)	HGU-133a	[140]
GSE6791	44 (30 + 14)	44 (30 + 14)	HGU-133plus2	[141]

### Merging the datasets and identification of DEGs

The COMBAT (empirical Bayes [33]) batch correction and cross-platform normalization method which is implemented in the inSilicoMerging package [34], was used to merge all normalized microarray datasets into one global merged dataset. The merging was performed by means of common identifiers to obtain common space across all platforms, with 22,277 genes in total. To assess the removal of the microarray bias effect across the datasets, another merged dataset without batch effect removal was also compiled. The datasets were projected onto the planes defined by the first two principal components. In addition, hierarchical clustering was performed using gplots software package. Ward’s method with the Euclidean distance metric was used for the clustering.In the biosvd [35] software package, the merged dataset was subjected to filtering out of eigengenes and eigenarrays—that were assumed to represent noise—by measuring steady-state gene expression and steady-scale variance. We performed nonspecific filtering using the library genefilter, to remove QC probe sets and genes with low overall variability as a standard procedure. Linear modeling and tests for differential expression, adjusted for multiple testing, were performed in the Linear Models for Microarray Analysis (LIMMA) [36] software package. Genes with a twofold higher or lower differential expression, and a false discovery rate (FDR) < 0.05 were selected as genes differentially expressed in tumor samples compared to normal controls.

### Construction and validation of a coexpression network

The coexpression network analysis was performed in the Weighted Gene Correlation Network Analysis (WGCNA) [37] software to identify modules of highly correlating genes. After initial data preprocessing for network analysis and removal of outliers, 347 tumor samples, which were further categorized into early- and late-stage samples, were used to construct a signed network, i.e., a network that preserves the sign of correlations among expression profiles. WGCNA defined the network into color-coded modules assigned according to the size (number of genes) with each module containing a set of unique genes. To assess the robustness of this definition of a coexpression module and to test whether the resulting modules were of high quality (rather than generated by chance), we examined their reproducibility by a resampling procedure [38]. Two methods to generate Z-summary scores were used. First, module statistics of the merged dataset (reference dataset) was compared to the randomly generated modules in a test dataset, which comprised 100 random samples from the reference dataset. Second, we also replicated module preservation analysis over individual GEO datasets by assuming that they were test datasets. Further, to incorporate the OSCC phenotype status into the coexpression network and identify stage-associated modules, we tried to find correlation of each module with disease phenotype. Modules that significantly correlated with stage phenotype were labeled “candidate modules”. In our analysis, only one module (pink) was found to correlate with a stage and therefore was analyzed further. The gene coexpression network for the candidate module was then visualized by importing network data into Cytoscape, version 3.0.1 [39]. To adjudge the scale-free nature of degree distributions of network, discrete power-law hypothesis was tested using poweRlaw software package [40]. Detailed information on the construction of coexpression network is provided in Supporting Information (Additional file 1: Supplementary Methods).

### Assessment of topological robustness of significant module(s)

This procedure was conducted by performing analyses of simultaneous node deletions [41] and by observing changes in the size of the largest component, σ(ρ), when the fraction ρ of vertices (nodes) was removed in a sequential manner. Importance of nodes was determined by first calculating critical topological centrality measures [42], including degree (*k*), betweenness centrality (BC), closeness centrality (CC), and eigenvector centrality (EC), for all nodes and then removing a certain fraction (ρ) of the nodes. Nodes were removed in the decreasing centrality order, consecutively followed by removal of nodes uniformly at random and by examination of changes in the size of the largest component. For the sake of simplicity, the network was assumed to be unweighted. The vulnerability of the network to a given scheme of vertex removal was quantified by computing the *V-*index, a value complementary to the *R*-index (*R*).

$$ V=\frac{1}{2}-R $$

### Identification of cancer hub genes and enrichment analysis

An ensemble of gene significance (*GS*_*i*_GS) and intramodular connectivity (*K*_*i*_) were considered to identify hub genes within cancer stage-associated module. Intramodular hub genes were selected based on stronger correlation with an OSCC stage (*GS*_*i*_>0.2) and higher connectivity (*K*_*i*_ > 0.3). *GS*_*i*_ describes strength of a correlation between a gene and a phenotypic trait. The higher the *GS*_*i*_GS, the stronger the gene’s absolute correlation with the trait of interest is. Conversely, intramodular connectivity was computed from the sum of its connection strengths with all other genes in the same module; this parameter is also called “scaled connectivity” (*K*_*i*_). *K*_*i*_ is measured as follows:

$$ {K}_i=\frac{k_i}{k_{\max }} $$

where *k*_*i*_ is the connectivity of a gene, and *k*_*max*_ is the maximal connectivity of the gene.

For enrichment analysis, Signaling Pathway Impact Analysis (SPIA) was performed on the hub genes using an ensemble of SPIA [43] and GRAPH Interaction from pathway Topological Environment (GRAPHITE) [44] software packages that predicted possible functional pathways dysregulated in OSCC. Next, gene ontology (GO) enrichment analysis was performed by the standard hypergeometric test from the GOstats [45] software package and Gene Ontology Consortium database [46], to identify categories of statistically over-represented biological processes (BP). To facilitate the interpretation and visualization of significantly enriched GO categories (*p* < 0.01), we used Reduce and Visualize Gene Ontology (REVIGO) (http://revigo.irb.hr/) [47].

### Construction of the stage classification model

The multivariate R package glmnet [48] was used to perform elastic-net feature selection by linear regression modeling. During this procedure, Classification And REgression Training (CARET) [49] package was set up to fine-tune both regularized parameters: α, the elastic net mixing parameter, and λ, the tuning parameter. After 1000 bootstraps, a frequency (*f*) ranked gene list was obtained on the basis of how often a gene was included in each bootstrap. Final hub gene signatures of the phenotype consisted of genes that were present in a >90th percentile cut off of the fourth quartile (750) of all bootstrap samples. All relevant features with non-zero coefficients were retained and assumed to be “key hub genes”. To assess classification power of the identified genes, a model was built in the glmnet software package. The model construction was based on 70 % of the training data, while model evaluation was performed on the remaining 30 % of testing data, with samples categorically classified into an early and late stage. Training was carried out by 5-fold cross-validation (each dataset was bootstrapped 5 times). The resulting model was then evaluated on the corresponding testing dataset.

Predictive performance was quantified by means of area under the receiver-operating characteristic (ROC) curve (AUC) by plotting the ROC curve in the pROC software package [50]. Furthermore, the classification model that was developed from the merged dataset was also evaluated on an independent dataset, and prediction power was further assessed. All computations were carried out on a 12-core HPZ600 workstation running the Ubuntu 12.04 operating system.

## Results

Our analysis consisted of several steps: a flow chart of the method is provided in Fig. 1. The results of our analysis are summarized in the following major steps: (1) data acquisition and merging of multiple microarray datasets to identify DEGs in tumor samples, (2) analysis of the gene coexpression network to identify cancer stage-associated modules, and (3) identification of cancer hub genes and development of a key hub gene-based classifier model to distinguish OSCC stages. Details of each step are summarized below.

### Data pre-processing

A total of six relevant experiments were obtained and five of them (five experimental designs) were explicitly dedicated to OSCC, whereas one was available for head and neck squamous cell carcinoma (HNSCC). A small sample size may result in unstable gene lists and poor prediction accuracy in studies on the differential gene expression [51]. Because the initial total number of samples obtained from six studies was small (191 cancer patients and 88 healthy (normal) subjects), an additional dataset (GSE30784) that lacked phenotypic details but contained a large number of OSCC samples (167 cancer patients and 45 healthy (normal) controls), was also included to ensure a sufficient sample size in this study. In the experiment involving HNSCC tissues, special care was taken to exclude non-OSCC samples. Hierarchical clustering analysis indicated that normal and tumor samples were grouped together with few outliers; however, separation between OSCC and normal groups was clearly observed. These observations implied that gene expression profiles of tumor samples were likely to be disordered in comparison with the healthy controls. Summary statistics of the fitted robust linear model was used to identify problematic chips: datasets GSE9844, GSE30784, GSE3524, GSE42743, and GSE2280 contained hybridization artefacts. Small localized artefacts may not be a cause for concern but some chips contained extensive defects: blemishes that affected most of the chip and therefore maximal number of probes. These chips were eliminated from further analysis as a standard procedure (Table 1).

Because we analyzed samples from multiple studies and thus from multiple arrays, the resulting gene expression data could be affected by batch effects: a non-biological experimental variation. The fRMA algorithm utilizes a platform-dependent background model to normalize the expression values [34]: therefore, the samples are clustered by platform and not by study. It was observed that normalisation introduced major deviating intensity levels into distributions with similar characteristics (Additional file 2: Figures S1 and S2). Furthermore, existence of any potentially defective arrays was also ruled out. The overall number of tumor (355) and non-tumor (131) samples was large enough to overcome the sample size restriction for differential-expression studies.

### Data merging and removal of the batch effect

**Fig. 2:**
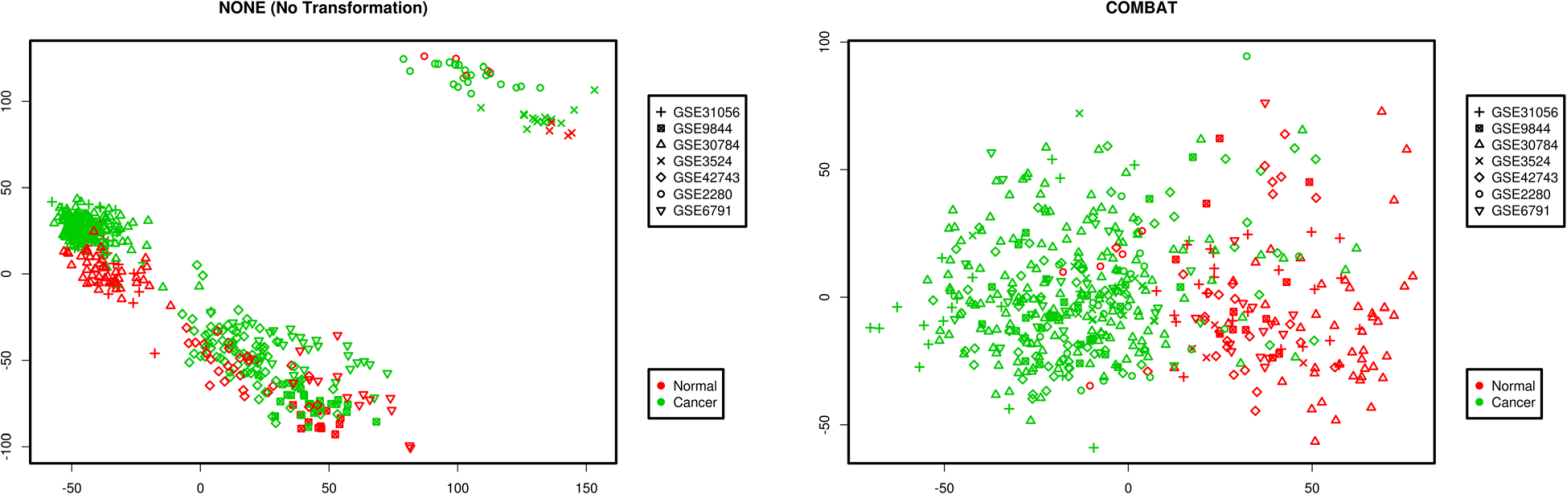
A multidimensional scaling (MDS) plot of the merged gene expression data.**a** This panel shows that without removal of the batch effect, all samples are clustered by experiment and by platform (not by the biological variable of interest) inside the MDS space. **b** With intra-platform batch adjustment, the samples are intermingled on the basis of the biological variable. All samples are color coded by biological variables (normal: red, cancer: green), with different symbols corresponding to different studies

Gene expression data that were generated by different processing facilities could not be combined directly for downstream analysis, even after processing with a similar normalization method. Many methods for batch effect removal have been developed [52], but COMBAT is believed to outperform other commonly used batch-adjustment methods [53]. In the present study, a COMBAT-merged dataset consisted of 22,277 genes in total, all of which mapped to distinct identifiers. The dataset was projected onto planes defined by the first two principal components via inspection of multi dimensional scaling (MDS) plots (Fig. 2). Principal components partition data into orthogonal linear components reflecting different contributions to variability in the data, with the first component explaining the largest contribution and the second component the second largest, and so on. To see impact of the batch effect, we also compiled another merged dataset without removal of the batch effect. Figure 2a shows that without additional transformation, all samples were clustered by experiment and by platform inside the MDS space and not by the biological variable of interest (normal versus tumor). This result indicated that the biggest source of variation was technical rather than biological. Inside each study, however, there was a clear separation between normal (healthy) and tumor samples. As expected, after implementation of intra-platform batch adjustment, the samples were intermingled on the basis of the biological variable (Fig. 2b); this situation means removal of dataset-specific biases and elimination of batch effects to a greater extent (though not complete elimination) and may facilitate clinically important analysis of the underlying biology.

Hierarchical clustering of the dataset comprising 1000 random genes was performed by Ward’s method with the Euclidean distance metric. The clustering analysis revealed that the samples were grouped and represented by a data source (Additional file 2: Figure S3-A); however, when they were simply combined, influence of the data source on the study-based grouping was significantly reduced after application of COMBAT (Additional file 2: Figure S3 B). Relative log expression (RLE) boxplots are proposed for validation of methods for batch effect removal [54] and were also used to illustrate a global bias between merged datasets. The RLE plot highlighted the existence of seven visible batches in simply combined datasets (Additional file 2: Figure S4 A), however COMBAT implementation improved the appearance of plot (Additional file 2: Figure S4 B). Additionally, compared to simply combined dataset (Additional file 2: Figure S4 A), the mean of the RLE plot for the COMBAT-processed dataset (Additional file 2: Figure S4 B) was distributed around zero and had almost similar spread for all samples; this situation was indicative of effective removal of the batch effect. These analyses clearly showed that cross-platform comparison outperformed the simple combining; therefore, the merged dataset was eventually used for further downstream analysis.

### Identification of DEGs

Filtering out of eigengenes and eigenarrays that are assumed to represent noise enables meaningful comparison of gene expression across different arrays in different experiments [55]. Low entropy (0.02) in combination with steady-state expression (~98 %) that were detected in the first eigenfeature suggested that the underlying processes were manifested by weak perturbations of the steady state of expression, and this eigenfeature was therefore filtered out. In contrast, no steady-scale variance was present in the dataset.

Multiple eigenfeatures were required to explain most of the variance; this result was indicative of the presence of various interesting signals. The core of LIMMA is an implementation of the empirical Bayes linear modeling approach, and provides more stable inferences about differential expression. Evaluation of the log ratio between conditions and consideration of genes that differ by more than an arbitrary cutoff represent reliable method for identifying DEGs [56]. It is also common to select those DEGs that satisfy both the p-value and fold change criteria simultaneously because this combination typically results in more biologically meaningful sets of genes [57–59]. In the present work, genes that simultaneously possessed low probability values and a high log-fold change were selected for analysis, where the t-statistic computed probability values and fold change offered statistically stringent and possibly biologically meaningful criteria, respectively. Of the 22,277 genes analyzed, a total of 1652 genes were found to be differentially expressed (1052 overexpressed and 600 underexpressed) (Additional file 2: Figure S5) thereby presenting evidence of variability between the case and control samples. To test the discriminative ability of the selected DEGs, we performed unsupervised hierarchical clustering of all samples on the basis of similarity in gene expression. A heatmap of the hierarchical clustering (Additional file 2: Figure S6) showed that most of the tissue samples were strictly clustered into their distinctly normal and cancer groups. This finding indicated that the DEGs that we identified could classify samples into their respective groups depending on gene expression patterns and therefore represented statistically significant genes.

### Identification of gene coexpression modules

**Fig. 3:**
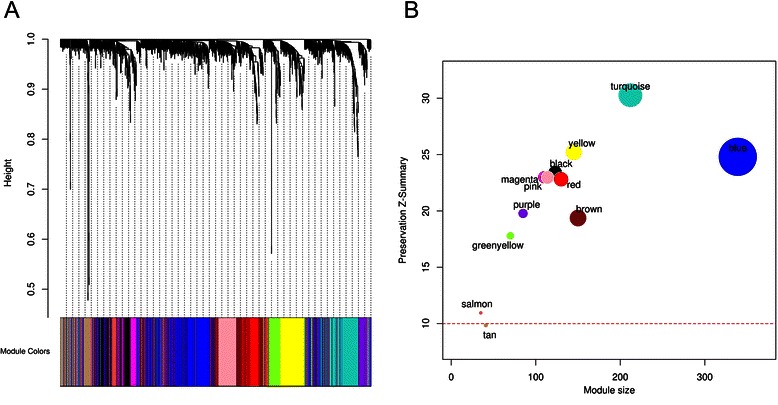
Module assignments for the expression data on oral squamous cell carcinoma (OSCC). **a** A gene dendrogram is constructed by average linkage hierarchical clustering. The color row underneath the cluster tree shows module assignment implemented by the dynamic tree cut method.**b** The Z-summary statistic (y-axis) of the original data modules against 100 random samples is plotted as a function of module size. Each circle represents a module labeled by a color and module name. The dashed redline denotes a significance threshold (Z = 10)

Because the transcripts that are involved in biological processes may be upregulated or downregulated, a signed network was created that allowed modules to contain positively and negatively correlated genes in different modules. WGCNA-weighted networks are highly robust with respect to the choice of the β value and provide an opportunity to construct networks with the scale-free criterion. To ensure biological relevance of the OSCC network, the β value of 18 was selected which resulted in a network that was approximately scale-free, and yielded the exceptionally high signed scale-free topology fit (R^2^) of 0.95. The node degree distribution for the network approximated a power-law distribution (Additional file 2: Figure S7), an inherent characteristic of scale-free networks, indicating the presence of few exceptionally connected genes in contrast to more frequent less connected genes. Hierarchical clustering defined branches of the cluster dendrogram in 13 color-coded modules (Fig. 3a) ranging in size from 35 to 339 genes (average size of 127 genes). Each module that we obtained was assigned to an arbitrary color according to size. Visual inspection of the gene dendrogram revealed modular organization since the genes whose expression highly correlated tended to cluster together in the same branches. It is worth noting that the grey module is always reserved for genes with dissimilar expression patterns that do not cluster into any other modules and therefore represent noise genes.

### Robustness and preservation of modules

Analysis of statistics of network module preservation determines whether modules that were identified in one network (reference) remain connected in another network (test) and yields a Z-summary statistic: a composite measure of statistics related to network density and connectivity. A module shows no evidence of preservation among datasets if its Z-summary statistic is smaller than two, whereas a statistics between two and 10 is corresponds to moderately preserved (reproducible) module, and above 10 is to a strongly preserved module [60]. While analyzing modules against 100 random samples from the merged dataset, we found that module preservation (mean Z-summary scores) ranged from 6.86 to 30.27 (Fig. 3b). Except for “tan”, which was moderately preserved, all modules were found to be above the significance threshold of 10. Module preservation was also fairly consistent across the majority of the bootstrapped networks obtained from an individual contributing dataset (Additional file 3: Table S1). We concluded that the majority of candidate modules can be considered moderately to highly reproducible. These results indicated that the modules identified were not dataset specific, but robust and highly reproducible structures.

### Analysis of modules associated with cancer stage

**Fig. 4:**
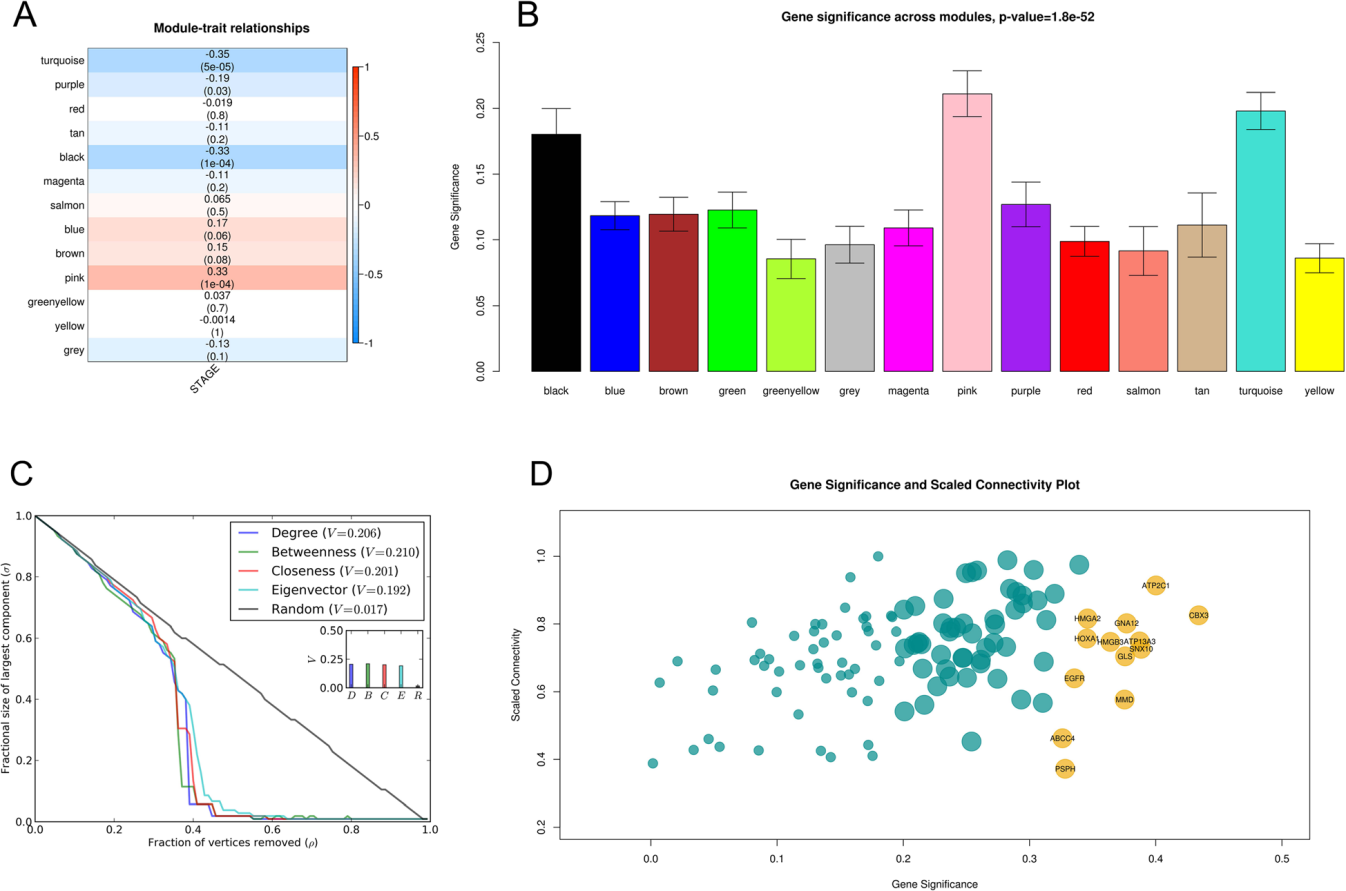
Analysis of expression data on oral squamous cell carcinoma (OSCC) in the WGCNA software. WGCNA: Weighted Gene Correlation Network Analysis. Suitability of the pink module is clearly visible.**a** A heatmap of module eigengenes (MEs) and correlations, where each row represents a module (labeled by color), and each column represents a trait. The value at the top of each square represents Pearson’s correlation coefficient between the MEs and trait, along with the associated p-value in parentheses. The red and blue colors represent a strong positive and negative correlation, respectively, between a ME and a trait. **b** Module significance (MS) of all modules, with pink at the top of the plot, indicating that expression profiles of the pink module are strongly associated with the trait. **c** Analysis of topological robustness of the pink module via plotting of a simultaneous node deletion against changes in the size of the largest component, σ(ρ), when the fraction ρ of the vertices (nodes) was removed. The results indicate network robustness. **d** The plot of gene significance (*GS*_*i*_GS) against scaled connectivity (*K*_*i*_) where each point (“darkgolden” and “darkcyan”) corresponds to a gene in the pink module. Intramodular connectivity significantly correlated with gene significance (*r* = 0.36, p = 8.3 × 10^−5^). All large labeled nodes (*GS*_*i*_ >0.2 and *K*_*i*_ > 0.3) are the identified hubs. Among these, darkgolden nodes represent hubs with the strongest correlation with the phenotype (*GS*_*i*_ >0.2, *K*_*i*_ > 0.3, and *f* >675); these hubs represent “key hub genes”

**Fig. 5:**
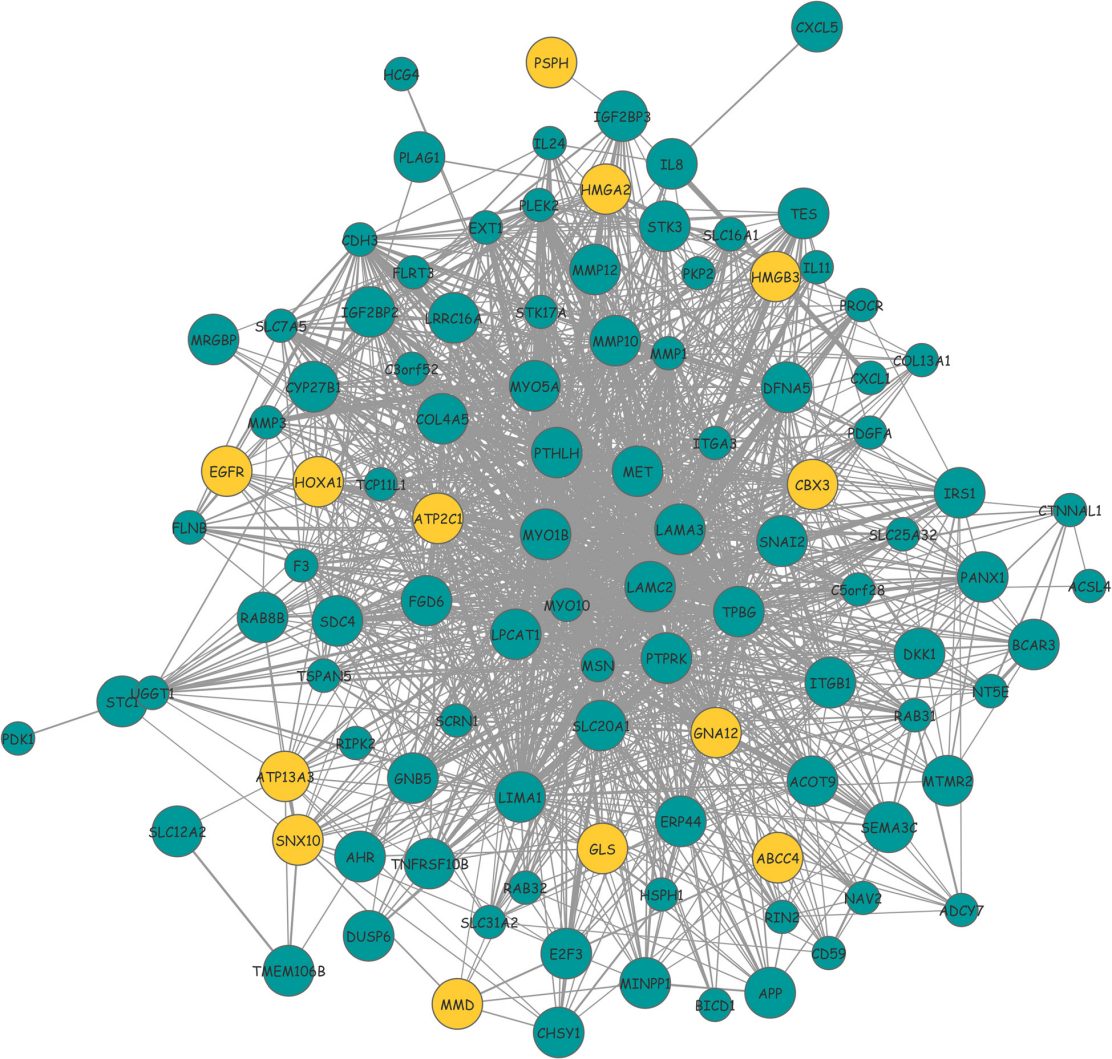
Visualization of hub genes in the pink module network. All gene-to-gene correlations were selected in the pink module, and the network was visualized by means of the Cytoscape software. Edge (grey) width is proportional to the weight of the correlation between two genes. All large labeled nodes are the identified hubs (gene significance [*GS*_*i*_GS] >0.2 and scaled connectivity [*K*_*i*_] > 0.3), whereas darkgolden nodes represent hubs that show the strongest correlations with the phenotype (these are “key hub genes”)

In comparison with the analysis of correlations of individual genes with clinical traits, module eigengenes (MEs) offer a major advantage because they reduce multiple testing from thousands of transcripts to the number of modules. By analyzing correlations of MEs with a stage phenotype, we obtained the most significant associations and identified groups of genes with strong relationships with an OSCC stage (Fig. 4a). For each correlation, p-values were computed and multiple testing corrections were performed using the Benjamini & Hochberg method for calculation of the FDR adjusted p-value (q-value). Among the 13 resulting modules, MEs that significantly correlated with phenotypic data of cancer stage were labeled “candidate modules” at the defined cutoffs (absolute correlation [*r*] ≥0.3 and *r* ≤ −0.3). ME of the pink module was found to positively correlate (*r* = 0.33, *p* = 5.5 × 10^−04^) with the stage; this module contains mostly those genes that were overexpressed at the cancer stage in question. In contrast, MEs of the black module (*r* = −0.33, *p* = 5.5 × 10^−04^) and turquoise module (*r* = −0.35, p = 5.5 × 10^−04^) correlated with the stage negatively; therefore, this result indicated that the genes in these modules had low transcript levels at the cancer stage in question. Expression patterns for each significant module were also unique (Additional file 2: Figure S8). In an attempt to find correlations of significant modules with gene expression profiles, we also defined *GS*_*i*_ and module significance (MS) network metrics. *GS*_*i*_ is the absolute value of correlation between a gene and a phenotype, and MS is average absolute *GS*_*i*_GS for all genes in a particular module. Figure 4b shows that pink, black, and turquoise modules stood out (had the highest MS), with pink at the top. Possible marker genes are specifically upregulated in the majority of tumors [61–63] and hence can be used to classify cancers according to stage [64]. The pink module (114 genes) was therefore finally selected on the basis of the strongest positive correlation with a stage and slightly higher module significance in comparison with the black and turquoise modules. The strong correlations suggested that the genes in the pink module contribute to or are driven by cancer stage. Visual representation of the pink module network is provided in Fig. 5.

### Topological robustness of the stage-associated module

Scale-free networks are prone to fragmentation under targeted attacks, and a decrease in network size, relative to the original size, represents inability of many partners to interact. Thus many biological processes are affected. It is apparent from the figure (Fig. 4c) that the pink module network almost precisely followed similar trends of vulnerable to a simultaneous targeted attack according to all centrality measures, thus reflecting their almost equal topological contribution to network integrity. Nevertheless, random (i.e., non-targeted) attack was much less effective at degrading the network structure. Similar effects of removal of hub nodes and removal of nodes with large BC values can be attributed to the fact that BC nodes are correlated with hub nodes [65], and are centrally located from the network point of view. A similar conclusion may also be drawn about other centrality measures; this situation is indicative of their advantageous locations in the network allowing them to act as intermediaries. The size of the largest connected component reduced gradually and reached zero after removal of ~40 % of nodes. Existence of a connected component until such a large percentage of nodes was removed is indicative of network robustness. Because central nodes were found to be responsible for network integrity, owing to the promiscuous interactions, these nodes may be useful for biological interpretations.

### Identification of cancer hub genes

Genes with significant interaction partners, also called hubs, are frequently found among existing cancer therapeutic targets and offer a promising approach to identify key genes. In this study, we assumed intramodular connectivity because it is far more meaningful than whole-network connectivity. Highly connected hub nodes are central to the network architecture [66] but may not always be biologically significant [67]. In practice, a combination of *K*_*i*_ and *GS*_*i*_ prioritizes genes that not only are central in network but also have phenotypic significance. A significant positive correlation (*r* = 0.36, *p* = 8.3 × 10^−05^) for such a small number of genes indicated that genes with higher connectivity tend to have a stronger association with cancer stage. Cancer genes often function as network hubs that are involved in many cellular processes [68], and play a pivotal role in the underlying mechanisms of disease. A total of 63 hub genes were obtained as reasonably good representatives of the pink module at the raised cutoff, and this number constituted ~55 % of all genes in the module (Fig. 4d). If eigengenes can explain a relatively large part of total variance of gene expression levels, then the subset of genes is considered important. Hub genes accounted for ~43 % of variation in the pink module eigengene; this result is relatively good, given that OSCC is a complex trait.

To find relations of known associations between the identified hubs and cancers, we obtained a list of genes—for which mutations have been causally implicated in cancer—from the Catalogue of Somatic Mutations in Cancer (COSMIC) database [69]. A list of tumor suppressor genes was also retrieved from the Tumor Suppressor Genes Database (TSGene) [70]. Not surprisingly, hubs included many genes that were already known to be involved in cancers. Of the 63 hubs, seven are annotated as well-known tumor suppressor genes, including *MET*, *DUSP6*, *DKK1*, *TES*, *ITGB1*, *PTPRK*, and *TNFRSF10B*. It is worth noting that somatic mutations in seven hub genes—*EGFR*, *MET*, *MYO5A*, *PLAG1*, *PTPRK*, *SDC4*, and *HMGA2*—have been implicated in cancer. Additionally, by means of data from other studies, various hub genes were found to be directly implicated in either OSCC or HNSCC, including known roles in other cancers (Additional file 3: Table S2).

### Analysis of the hub genes for functional pathway enrichment

**Fig. 6:**
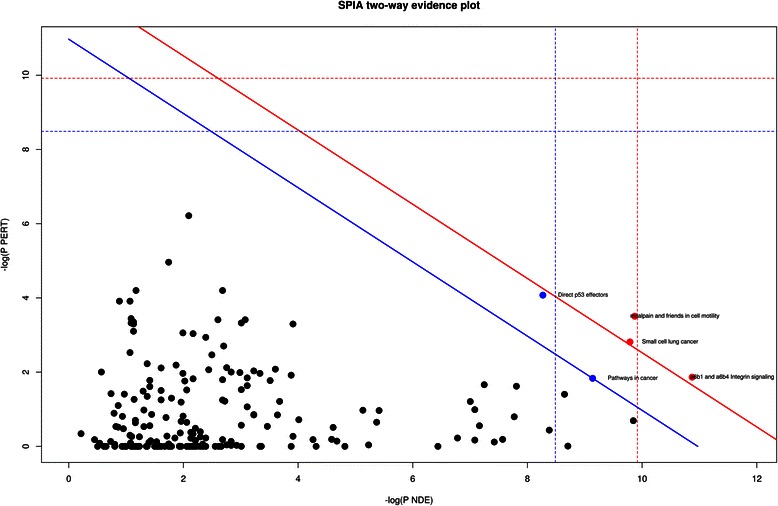
Significantly enriched pathways among the hub genes. A two-way evidence plot of signaling-pathway impact analysis (SPIA) for each pathway is represented by one dot. Pathways on the right of the red oblique line (red dots) are statistically significant at the 1 % threshold after Bonferroni correction of global p-values (P_G_) obtained by combining (by Fisher’s method) over-representation of differentially expressed genes (DEGs) in a given pathway (P_PERT_) and an abnormal perturbation of the pathway (P_NDE_). The pathways on the right of the blue oblique line (blue dots) are statistically significant after false discovery rate (FDR) correction of P_G_

Hub genes are thought to be candidate drivers of a module; therefore, elucidation of their associated pathways should provide insights into the altered biological mechanisms in a diseased condition. Many of the existing pathway analysis methods are focused on either the number of DEGs in a pathway or on the correlation among genes in the pathway [71], thus disregarding the information about complex gene interactions. SPIA takes into account the information from a set of DEGs and their fold changes as well as a pathway topology in order to assess the significance of pathways and to obtain a global probability value (P_G_). P_G_ is obtained by combining P_NDE_ and P_PERT_ by Fisher’s method where P_PERT_ and P_NDE_ are the over-representation of DEGs in a given pathway, and abnormal perturbation of that pathway, respectively. Pathways significant at the 1 % threshold after the Bonferroni- and FDR-corrections of P_G_ are shown as red and blue data points, respectively, in Fig. 6. SPIA revealed five significantly perturbed (*p* <0.01) pathways, including two activated (“*small cell lung cancer*” and “*pathways in cancer*”) and three inhibited pathways (“*direct p53 effectors”*, “*mcalpain and friends in cell motility”*, and “*a6b1 and a6b4 integrin signaling”*; Additional file 3: Table S3). Overall, these pathways mediate cellular processing, signal transduction and cancer mediated processes. Activation of the “*pathways in cancer”* and “*small cell lung cancer”* pathways can be directly attributed to OSCC progression.

The p53 protein regulates the cell cycle and functions as a tumor suppressor [72], and inhibition of p53’s regulatory elements leads to dysregulation of various tumor-suppressing processes, including DNA repair, cell cycle arrest, senescence, and apoptosis. Moreover, p53 is also the most frequently mutated gene in oral cancer [73]. Calpains have been shown to play a pivotal role in cancer development and progression, cell transformation, tumor invasion, apoptosis, angiogenesis [74], and cell migration [75],which is a critical step in tumor invasion and metastasis. The m-calpain is also required for growth factor receptor-mediated de-adhesion and motility [76]. Activity and protein expression of m-calpain are significantly elevated in cancers [77], but paradoxically this pathway was found to be inhibited.

Numerous studies have shown altered integrin expression profiles during cancer growth and progression, and this kind of changes contribute to the aggressive behavior of cancer cells [78]. Moreover, the involvement of α6β4 integrin in cancer progression has been well elucidated [79], but few reports described the role of α6β1 integrin in tumor progression [80]. Furthermore, it is known that dysfunctional integrin signaling is involved in the detachment of tumor cells from neighboring cells, ensuring enhanced survival and proliferative abilities [81]. Altogether, our results indicated that the pink module may also be considered as an oncogenic one because it is enriched in well-known cancer-related pathways.

### Analysis of the hub genes for gene ontology enrichment

To obtain functional annotation of gene products, GO BP defines biological events to which a gene or gene product contributes [46]; therefore, enriched GO BP terms tend to provide insights into functional characteristics of genes. For hubs to be of significant relevance, they would have to carry ontological signatures specific to cancer. We found that major significantly enriched so-called supercluster terms were related to molecular mechanisms associated with a “*polyphosphate metabolic process*” (GO:0006797), “*cellular response to vitamin D*” (GO:0071305), “*mitotic G2 phase*” (GO:0000085), “*regulation of single stranded viral RNA replication* via *double stranded DNA intermediate*” (GO:0045091), “*negative regulation of keratinocyte proliferation*” (GO:0010839), and “*gland morphogenesis*” (GO:0022612; Table 2). Enrichment in the *polyphosphate metabolic process* may be directly related to the enhanced metabolic activity and energy consumption rate. Polyphosphate (polyP) performs an important role in apoptosis and enhancement of mitogenic activity of fibroblast growth factor [82], but polyP’s precise role is poorly understood. Phosphate can act as a signaling molecule on the extracellular signal-regulated kinase (ERK1/2) [83] and adenylate cyclase/cAMP signaling pathways [84], and can ultimately affect cell growth. PolyP levels are also found to be increased in glioma and lung cancer cells [85].

**Table 2:** Categories of functionally enriched gene ontology (GO) biological processes (BPs) in the pink module. The latter is the cancer-associated module, and hub genes from this module are shown in the table

Representative GO term	BP ID	Frequency (*%*)	Hypergeometric p*-*value	Genes in GO category
*polyphosphate metabolic process*	GO:0006797	0.001	5.789 × 10^−03^	*MINPP1*
*cellular response to vitamin D*	GO:0071305	0.040	2.153 × 10^−05^	*AHR, APP, ATP2C1, CXCL5, CYP27B1, DFNA5, DKK1, DUSP6, EGFR, FGD6, GLS, GNB5, HMGA2, HOXA1, IL8, IRS1, ITGB1, MET, MTMR2, MYO5A, PANX1, PSPH, PTHLH, PTPRK, RAB8B, SLC12A2, SLC20A1, SNAI2, STC1, STK3, TNFRSF10B*
*mitotic G2 phase*	GO:0000085	0.013	1.156 × 10^−03^	*APP, E2F3*
*regulation of single stranded viral RNA replication* via *double stranded DNA intermediate*	GO:0045091	0.009	4.873 × 10^−04^	*AHR, APP, ATP2C1, CYP27B1, DFNA5, DKK1, DUSP6, E2F3, EGFR, FGD6, HMGA2, HOXA1, IL8, IRS1, ITGB1, LPCAT1, MET, MTMR2, PANX1, PLAG1, PTHLH, RAB8B, RSF1, SDC4, SLC20A1, SNAI2, STC1, STK3, TNFRSF10B*
*negative regulation of keratinocyte proliferation*	GO:0010839	0.015	4.873 × 10^−04^	*APP, CXCL5, E2F3, EGFR, HMGA2, IRS1, ITGB1, MMP12, PLAG1, PTHLH, PTPRK, SNAI2*
*gland morphogenesis*	GO:0022612	0.399	2.716 × 10^−05^	*AHR, APP, ATP2C1, CHSY1, COL4A5, CYP27B1, DFNA5, DKK1, DUSP6, EGFR, FGD6, GNA12, HMGA2, HOXA1, IL8, IRS1, ITGB1, LAMA3, LAMC2, LPCAT1, MET, MINPP1, MMP10, MMP12, MTMR2, MYO5A, PLAG1, PTHLH, SEMA3C, SLC12A2, SNAI2, SNX10, STC1*

Some processes were also found to be altered in relation to vitamin D stimulus (*cellular response to vitamin D*). Vitamin D receptor (*VDR*)—a key mediator of the vitamin D pathway—has been implicated in insulin-like growth factor signaling, inflammation, estrogen-related pathways, and activation and regulation of vitamin D and calcium. Involvement of *VDR* in multiple pathways and points of convergence within these pathways indicate its possible importance for etiology of cancers [86]. Furthermore, polymorphisms in the *VDR* gene are associated with prostate cancers; this finding supports the role of *VDR* in the risk of some type of cancers [87, 88]. Therefore, in theory, there may be dysregulation of processes or genes involved in the pathways associated with *VDR*.

Because cancer cells continue to reproduce indefinitely, as expected, biological activities are also significantly altered in cell cycle regulation. The enriched term *mitotic G2 phase* [89] is known to be directly linked to tumorigenesis and progression of cancer [90], contributing to a faster cell cycle during tumor growth. Some types of oral cancers are linked to human papilloma virus (HPV). Although epidemiology of oral HPV infection is not fully understood and its prevalence and importance are controversial, the enriched term *regulation of single stranded viral RNA replication* via *double stranded DNA intermediate* [91] may point to the altered processes due to HPV infection [92]. The GO term *negative regulation of keratinocyte proliferation* related to processes associated with multiplication or reproduction of keratinocytes; these processes ultimately increase the cell population. Malignant oral keratinocytes express 5–50 times more *EGFR* than do their healthy counterparts [93]; therefore, activation of *EGFR* enhances proliferation and the metastatic potential of keratinocytes [94].

The enriched term *gland morphogenesis* possibly indicates alteration of some processes during salivary gland neoplasia [95] as it is an outcome of modified morphogenetic events. Other noteworthy BP terms, including their child terms, are provided in Additional file 3 (Table S4). Non redundant BP terms were subsequently visualized as a tree map (Additional file 2: Figure S9). Since GO terms revealed significant enrichment in processes implicated in cancer progression, this finding provided evidence that hub gene-mediated processes were significantly dysregulated during OSCC progression.

### Regularized logistic regression modelling

Among the hub genes selected, some may be irrelevant to the trait of interest; therefore, we performed multivariate logistic regression modeling to reduce the dimensionality of the feature space and to identify the best subset of hub genes (those that have the strongest correlations with the phenotype in question). The elastic net, an automatic method of variable selection, interpolates between *L1*- (LASSO) and *L2*- (ridge) regularization and can effectively shrink coefficients and set some coefficients to zero. After running 1000 bootstraps, we obtained a frequency (*f*) ranked gene list based on how often a gene was included in each bootstrap. The optimal subset of features consisted of genes that were present at a greater-than-selected threshold (*GS*_*i*_ >0.2; *K*_*i*_ >0.3; *f* > 675) and therefore were considered to be “key hub genes” in this study. Finally, 13 key hub genes—*CBX3*, *PSPH*, *ATP2C1*, *SNX10*, *MMD*, *ATP13A3*, *GLS*, *EGFR*, *GNA12*, *ABCC4*, *HMGB3*, *HMGA2*, and *HOXA1* (Table 3)—were consistently identified as important above the selected thresholds. It is noteworthy that many of the selected genes have well known cancer associations (see Discussion). Visual representation of key hub genes in the pink module network is provided in Fig. 5.

**Table 3:** Key hub gene signatures based on an ensemble of centrality and trait relevance criteria (Gene significance [*GS*_*i*_GS] >0.2, scaled connectivity [*K*_*i*_] 0.3, frequency [*f*] > 675)

Entrez ID	Approved Gene Symbol	Approved Gene Name	Scaled connectivity (*K*_*i*_)	Gene significance (*GS*_*i*_GS)	Frequency (*f*)
11335	*CBX3*	chromobox homolog 3	0.82	0.43	982
5723	*PSPH*	phosphoserine phosphatase	0.37	0.32	980
27032	*ATP2C1*	ATPase, Ca++ transporting, type 2C, member 1	0.91	0.40	948
29887	*SNX10*	sorting nexin 10	0.72	0.38	936
23531	*MMD*	monocyte to macrophage differentiation-associated	0.57	0.37	936
79572	*ATP13A3*	ATPase type 13A3	0.74	0.38	929
2744	*GLS*	glutaminase	0.70	0.37	923
1956	*EGFR*	epidermal growth factor receptor	0.63	0.33	869
2768	*GNA12*	guanine nucleotide binding protein (G protein) alpha 12	0.80	0.37	840
10257	*ABCC4*	ATP-binding cassette, sub-family C (CFTR/MRP), member 4	0.46	0.32	770
3149	*HMGB3*	high mobility group box 3	0.74	0.36	765
8091	*HMGA2*	high mobility group AT-hook 2	0.81	0.34	744
3198	*HOXA1*	homeobox A1	0.75	0.34	676

**Fig. 7:**
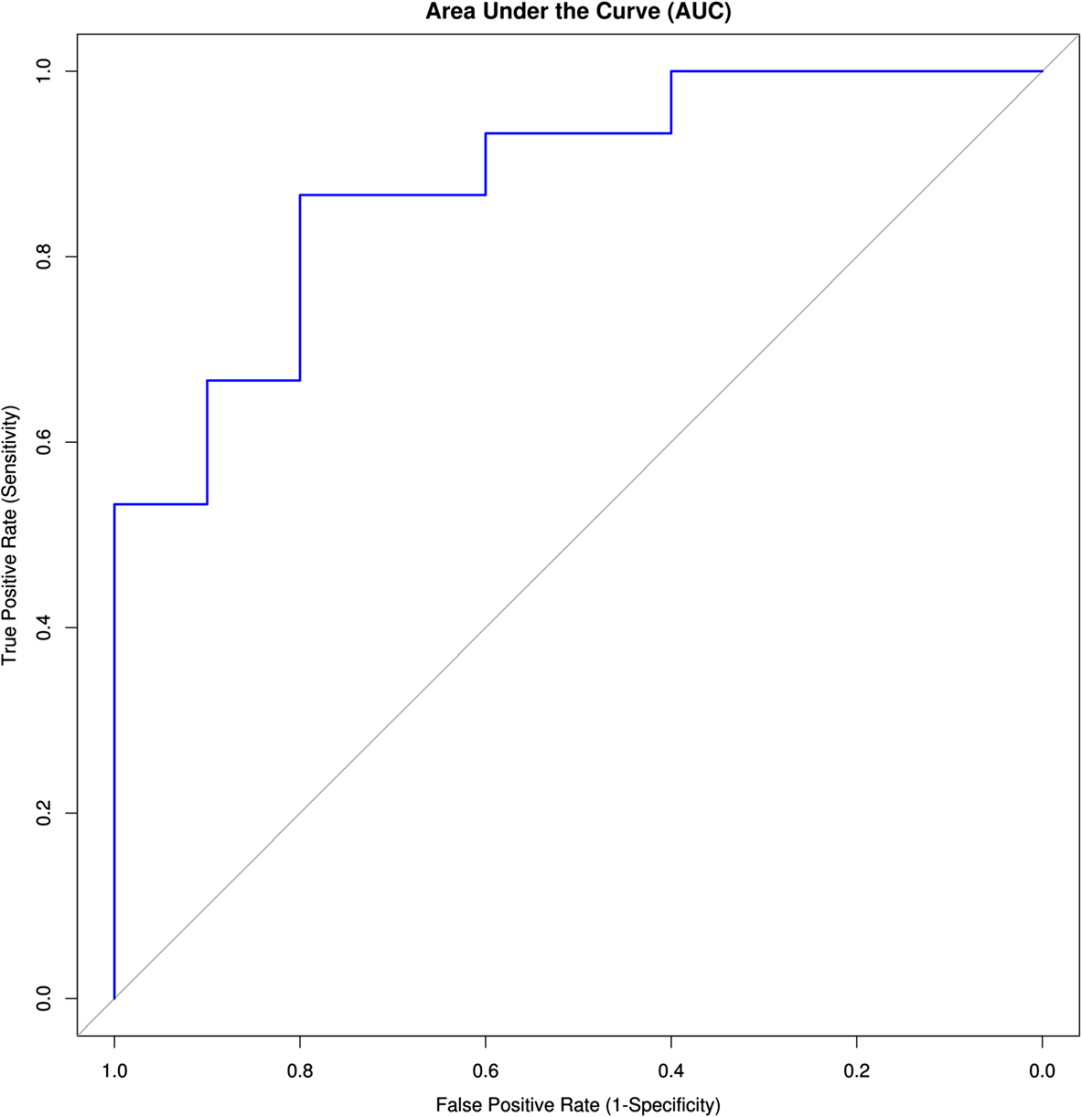
The plot of a receiver-operating characteristic (ROC) curve. The average area under the curve (AUC) of ~0.81 denotes the accuracy of the signature of key hub genes in the test dataset. The ROC curve depicts a true positive rate (sensitivity) versus a false positive rate (one minus specificity). The diagonal line in the ROC plot has an AUC value of 0.5, representing the predictive power of a random guess. The graph was rendered in the ROCR software

After performing feature selection, a classifier model was also built using the above-mentioned 13 genes to determine whether the identified candidate genes can discriminate between early- and late-stage OSCC samples. The classification accuracy (AUC) of the five generated models was found to be 0.88, 0.73, 0.85, 0.84 and 0.72, with the average of ~0.81 (Fig. 7). Since the average AUC value was greater than 0.50, the classification algorithm screening performed better than did random discrimination. Furthermore, the resulting prediction model was also evaluated on an independent dataset containing 54 late- and 41 early-stage OSCC tumor samples. The gene expression dataset (GSE41613) obtained was measured by means of an Affymetrix array containing expression and phenotypic data from HPV-negative OSCC samples (of a known stage) [96]. Using our model, we obtained a reasonable ~61 % accuracy in the independent dataset. Overall, these results indicated that gene features that we identified offered substantial predictive power for classification by phenotype-associated stages (early- versus late stage).

## Discussion

The clinical stage of cancer is the most important determininant of a treatment regimen for a patient and is useful for assessment of the risk of metastases and for prediction of recurrence and mortality in patients with oral cancer; thus, the cancer stage is an efficient tool for improvement of survival rates. Accordingly, to improve the survival rates among the patients, OSCC should be diagnosed as early as possible. Given the difficulty of procuring stages in cancer, it is important to determine expression profiles of genes associated with these stages. The emerging field of systems biology can help to elucidate biological mechanisms underlying complex traits and may provide a functional context for identifying those genes that contribute to cancer development. Alterations in gene expression correlate with a tumor histotype, grade, and stage. Various researchers have utilized differential expression analysis along with coexpression network mining and identified important regulatory networks in cancer datasets [97]. Furthermore, network-based approaches are known to perform better at prediction of cancer metastasis in comparison with gene-based approaches [98]. Considering these advantages, we implemented a meta-analysis and knowledge-independent approach to construct a gene coexpression network and then developed a stage prediction model to discriminate early- and late-stage OSCC tumors. Our assumption was that a group of interconnected genes with topological and trait relevance (rather than a large number of individual genes) may yield most reliable predictions regarding an OSCC stage.

Thus, the WGCNA-based gene coexpression network was analyzed here to identify modules of strongly correlated genes during OSCC stage progression. Although many alternative coexpression network methods have been proposed [99], WGCNA offers several valuable opportunities including interpretation of module robustness, calculation of network properties, and the possibility of association of modules with external clinical traits. In this study, the network was based on analysis of tumor samples; therefore, it can provide a glimpse into the disease status where specific characteristics of stage progression are involved. Because modules showed genes of biological interest, focusing on them seems to be a biologically meaningful data reduction scheme. One coexpression module (pink) was found to be strongly associated with cancer stages (Fig. 4a), suggesting that genes in this module contribute to or are driven by a cancer stage. Furthermore, the pink module is also distinct from the others in the sense that genes within this module positively correlate with the phenotype of an OSCC stage and offer the highest significance of the module as well (Fig. 4b).

Gene regulation occurs in the context of complex networks of interactions among multiple genes rather than in a linear one-to-one process [100]. Much attention has been focused on the modular approach to selection of targets for a therapeutic intervention. In the present study, we further refined the core of oncogenic module by identifying the most central genes also called hubs. It is worth noting that the number of hubs depends upon the threshold and can be varied accordingly. Modules may arise due to non-biological variation; therefore they were tested for enrichment.

As mentioned earlier, different cancers can share common characteristics including cell cycle regulation [90], phosphate metabolic processes [84], and regulation of keratinocyte proliferation [93] (Table 2).Our findings are consistent with the notion that these common processes are associated with cancers.

Additionally, ontological associations of hub genes can be extended to a neighboring highly connected gene cluster comprising coexpressed genes, within the confines of a given module. Besides categories related to cancer-associated ontological terms, these central players showed direct significant evidence of known cancer-associated pathways. Several of the hub genes that we identified in the pink module are consistent with the results of other studies on cancer, though some hub genes did not show any association with OSCC. Apart from their topological centrality, gene significance—which measures the strength of differential gene expression between the early- and late-stage groups—also points to an important association of these genes with the phenotypic traits of an OSCC.

A smaller number of biomarkers often does not perform well, whereas a cancer classification system that is based on an expression profile of a reasonable number of genes can outperform standard systems that are based on clinical and histological criteria [101, 102]. Feature screening is a useful approach to analysis of multidimensional data with the aim of identifying all features relevant to the response variables. Elastic-net regression has been proposed as a way to select significant features and was even used to select genes relevant to diagnosis or prognosis of a disease [103, 104]. In our analysis, elastic-net regression modeling was used to identify the best subset of hub genes (those that have the strongest correlations with the stage phenotype). The classifier model that was developed by means of differential expression of 13 key hub genes was potent enough to discriminate between OSCC stages.

Detailed and systematic literature search suggests that dysregulation of these hub genes is directly involved in OSCC and may play an important role in the development of other cancers. For example, *GNA12* [105], *GLS* [106], *SNX10* [107], and *HOXA1* [108] are often dysregulated in OSCC, while the genes *CBX3* [109] and *ABCC4* [110] are differentially regulated in HNSCC. In addition, *CBX3* and *ABCC4* are promising therapeutic targets in osteosarcoma [111] and pancreatic cancer [112], respectively. *CBX3* has been reported to function in chromatin packaging and gene expression regulation and has also been found to possibly regulate euchromatin repression by associating with nucleosomes in heterochromatin [113]. Some studies have also shown that high expression of *CBX3* is associated with other tumors [113].

Up-regulated genes *EGFR* [114] and *HMGA2* [115] are possible tumor markers of OSCC. *HMGA2* was found to be highly expressed in metastatic lung adenocarcinoma and contributes to cancer progression and metastasis [116]. *EGFR* regulates signaling pathways that participate in developmental processes, including cell cycle activation, cell survival, proliferation, and angiogenesis [117]. Furthermore, dysregulation of *EGFR* is among the most frequently studied molecular events that leads to oral carcinogenesis, and OSCCs show upregulation of *EGFR* by 42 % to 58 % [118]. Various studies have highlighted the role of *EGFR* in the pathogenesis of oral carcinoma [119], and this protein is also frequently expressed in many types of cancer, including HNSCC [120].

In the present study, involvement of some of the selected genes in OSCC is not supported by direct evidence; however, they may be linked to tumorigenesis indirectly. For example, *GLS* plays an important role in cell growth and energy metabolism during cancer stage progression [121], while *HMGB3* promotes cell proliferation and migration and is associated with poor prognosis in urinary bladder cancer [122]. In addition to large consumption of energy, cancer cells accumulate building blocks like nucleic acids, lipids, and cofactors for construction of new cellular components, including amino acids [123]. Glutamine is among the main sources for maintenance of activity of essential metabolic pathways such as glycolysis and the anaplerotic flux of the tricarboxylic acid cycle. *GLS* converts glutamine to glutamate and performs an important function in cell growth and energy metabolism during cancer stage progression [121]. Furthermore, some reports have suggested that inhibition of *GLS* slows down the growth of glioblastoma cells and therefore may be therapeutic strategy against such cancers [121].

Although precise role of the *HMGB3* gene has not been determined in OSCC, this gene promotes cell proliferation and migration and performs an important function in DNA replication, recombination, and repair [122]. Altered expression of this gene is also associated with other cancers including urinary bladder cancer [122], metastatic breast cancer [124], and gastric cancer [125].

Metabolic processes can be adapted in a way to drive macromolecular biosynthesis for rapid cell growth and proliferation [126]. Serine can be converted to glycine, which provides carbon units for one-carbon metabolism; therefore, the serine biosynthetic pathway plays a crucial role in glucose conversion [127]. The *PSPH* gene was found to be upregulated in the L-serine biosynthetic pathway during metastasis [128]. Serine biosynthesis is also elevated in tumor lysates [129], where *PSPH* acts as a rate-limiting enzyme of this pathway [130]; this finding is indicative of its regulatory role in tumor progression.

Tumor-associated macrophages (TAMs) have both positive and negative effects on tumor growth of various cancers. Since TAM expression is significantly associated with stages of invasion, these cells possibly play a role in angiogenesis during oral cancer progression [131].

*MMD*, a gene associated with differentiation of monocytes into macrophages, is a key signature of a relapse and survival among patients and is involved in lung cancer [132]. Although function of the *MMD* protein is unknown, certain studies have shown that macrophage activation promotes cancer metastasis [133].

*GNA12* mRNA levels are significantly upregulated in OSCC, and consistently high levels of the *GNA12* protein expression are detected in ~75 % of OSCC tissues. Overexpression of this protein drives migration and invasion of oral cancer cells; targeting of *GNA12* was proposed for prevention of metastasis [105].

Although the expression profile and function of *ABCC4* in OSCC remain unclear, this gene is a promising target for treatment of pancreatic cancer[112]. *HOXA1* is dysregulated in various cancers, for example it is overexpressed in OSCC [108] (where it promotes cell proliferation) and is downregulated in small cell lung cancer [134].

MicroRNAs are important for regulation of post-transcriptional repression of some genes and have been identified as statistically unique markers for discrimination of cancer from healthy tissue, thus serving as a valuable tool for cancer diagnosis [10, 135]. Differentially expressed microRNAs have been found to tightly regulate four of the possible biomarkers that we identified—*HMGA2*, *EGFR*, *HOXA1*, and *ABCC5*/*ABCC4* [10]—further supporting their involvement in oral-cancer progression. Although the exact role of some of the selected genes—*PSPH*, *ATP2C1*, *MMD*, *ATP13A3*, *ABCC4*, and *HMGB3*—has not been established in OSCC, a modular hierarchy and “guilt-by-association” rule may be utilized to identify the direct (or indirect) association of these genes with OSCC. Because these hub genes have good predictive power at distinguishing OSCC stages, they may provide new insights into the biological mechanisms underlying the stage progression.

Our study was somewhat limited by the next-generation sequencing data perspective [136], and we used a relatively small set of genes in the coexpression analysis. Additionally, due to limited availability of clinical data, it was not possible to incorporate other phenotypic parameters into our analysis (e.g., cancer grade and type). Nevertheless, our study has several strengths including the use of multiple large datasets, careful QC, a powerful statistical approach to identification of modules, and a well established and validated model that can effectively discriminate between early- and late-stage OSCC tumors. Despite simplicity, our systematic analysis illustrates a method for classification of OSCC stages; this method can help researchers to identify the cancer stage by means of molecular features instead of histopathological analysis or measurement of tumor size. With the availability of additional samples and inclusion of more clinical and topological feature vectors, the accuracy of this prediction model may be enhanced. We expect that, if confirmed in empirical studies, the selected gene features will speed up the discovery of molecular signatures of stage progression in OSCC. This approach may also open up opportunities for development of novel diagnostic modalities or therapeutic interventions in other cancers.

## Conclusions

We present a systematic computational and statistical pipeline, comprising differential expression, analysis of a gene coexpression network, and logistic regression modeling. This method helped us to identify13 key hub genes as features associated to stage progression in OSCC. We were able to differentiate between early- and late-stage tumor samples on the basis of differences in expression profiles of the 13 identified genes. Although some of the hub genes are OSCC-specific, many have been implicated in other carcinomas. Since all genes in a module are strongly associated, our results may point to a number of other promising candidate genes that warrant further analysis; hence, empirical studies will be needed to address their specific roles. For example, the precise roles of some of our selected genes—*PSPH*, *ATP2C1*, *MMD*, *ATP13A3*, *ABCC4*, and *HMGB3*—are unknown in OSCC. If characterized, these genes may define OSCC-associated processes or may serve as possible therapeutic targets. The selected gene set may turn out to be a valuable reference set for identification and validation of biomarkers of OSCC. To the best of our knowledge, this study is the first implementation of key hub genes (by means of expression profiles and coexpression network) to develop a classification model for OSCC stages.

## Additional files

Additional file 1: 
**(Supplementary Methods).**
Additional file 2: Figure S1. (Supplementary Figures). Expression intensity distributions of arrays. Density plots of arrays of probe-level data before (A) and after (B) normalization. **Figure S2.**Expression intensity distributions of arrays. Box plots of arrays of probe-level data before (A) and after (B) normalization.**Figure S3.** Heatmap plot analysis of merged gene expression data without (A) and with (B) batch effect removal approach. The hierarchical clustering was performed by means of gplots software package. Ward's method with Euclidean distance metric was used for the clustering. The samples are displayed on the X-axis while genes are listed on the Y-axis. The vertical branches of cluster, representing samples, are color coded according to group with “dark-red” and “forest-green” for cancer and normal samples, respectively. The resulting heatmap shows that the samples are clustered together by data source when they are simply merged (A); however, after the application of COMBAT, the influence of data source on grouping is significantly reduced (B). This COMBAT-merged method resulted in clustering of the samples into their distinct normal and cancer groups. **Figure S4.** Relative log expression (RLE) boxplots. A plot indicating mean of RLE is not centred around zero and represent an uneven spread when they are simply merged (A); however, after the application of COMBAT, the mean of RLE plot was distributed around zero for all genes (B). This result is an indicative of removal of batch effect by COMBAT-merged method. Additionally, the plot highlighted the existence of 7 clear batches in simply combined datasets (A); however, COMBAT-implementation method greatly improved the appearance of plot (B). All samples are color coded based on biological variable of interest (healthy (red); cancer (green)). **Figure S5.** Volcano plot of differentially expressed genes (DEGs). The log2 fold change is plotted on the X-axis and the negative log10 adjusted p-value is plotted on the Y-axis. Cyan circles graphically display 1652 genes that satisfy both criteria of p-value (False Discovery Rate [FDR] < 0.05) and fold change (>two-fold change). **Figure S6.** Clustering analysis of differentially expressed genes (DEGs). The hierarchical clustering was in the gplots software package. Ward's method with Euclidean distance metric was used for the clustering. The clustering of DEGs shows a distinct separation between normal and cancer groups. The samples are displayed on the Y-axis and genes are listed on X-axis. The vertical branches of cluster, representing samples, are color coded according to group with “dark-red” and “forest-green” for cancer and normal samples, respectively. **Figure S7.** The node degree distribution of Oral Squamous Cell Carcinoma disease network. The number of nodes (i.e., genes) is plotted as a function of their degree which reflects a power-law like distribution; this is an indicative of scale-free network topology. The red line corresponds to a power-law distribution with parameters xmin = 44 and α = 6.62, where xmin is lower cut-off and α is the scaling parameter. **Figure S8.** The expression heatmap plots of modules correlated with a stage. The rows correspond to genes and the columns to random samples. Here, in this figure, genes color coded green are underexpressed, while red indicates overexpression. **Figure S9.** The enriched gene ontology (GO) terms obtained for hub genes. The enrichment analysis (Fisher exact test p < 0.05) is carried out by means of GoStats software package and summarized using REVIGO. Additional file 3: Table S1. (Supplementary Tables). The Z-summary statistics of modules obtained against bootstrapped networks from the individual dataset from Gene Expression Omnibus (GEO). **Table S2.** The roles of hub genes (corresponding to pink module) highlighting their significance in OSCC and other cancers. **Table S3.** The perturbed pathways obtained in hub genes on the basis of combined Signaling Pathway Impact Analysis (SPIA) criteria after a False Discovery Rate correction of 1 % on the global p-values (pG). (pSize: number of genes in the pathway; NDE: number of DE genes in the pathway; pNDE: Probability to observe at least NDE genes in the pathway using a hypergeometric model; tA: observed total perturbation accumulation in the pathway; pPERT: probability to observe a total accumulation more extreme than tA by chance; pG: p-value obtained by combining pNDE and pPERT; pGFdr: False Discovery Rate; Status: Direction in which the pathway is perturbed). **Table S4.** The REVIGO summarized biological process (BP) categories terms for hub genes (corresponding to pink module).

## Abbreviations

AUC: Area under the ROC curve
BC: Betweenness centrality
BP: Biological processes
DEG: Differentially expressed gene
FDR: False discovery rate
GEO: Gene Expression Omnibus
GO: Gene ontology
*GS*_*i*_GS: Gene significance
HNSCC: Head and neck squamous cell carcinoma
HPV: Human papilloma virus
MDS: Multidimensional scaling
ME: Module eigengene
MS: Module significance
OSCC: Oral squamous cell carcinoma
ROC: Receiver-operating characteristic
WGCNA: Weighted gene correlation network analysis
