# Supplementary material for: Integrated network analysis and logistic regression modeling identify stage-specific genes in Oral Squamous Cell Carcinoma
Source: BMC Med Genomics. 2015 Jul 16;8:39. doi: 10.1186/s12920-015-0114-0 (PMC4502639; doi:10.1186/s12920-015-0114-0)
Supplement: Additional file 1: — (Supplementary Methods). [file 12920_2015_114_MOESM1_ESM.docx]

**Additional File 1 (Supplementary Methods)**

**Supplementary Methods**

**1. Construction and validation of coexpression network**

**1. 1. Identification of modules**

The coexpression network analysis was performed in the Weighted Gene Correlation Network Analysis (WGCNA) [1] software package to identify modules of highly correlated genes during stage progression of OSCC. A signed network i.e., a network that allows the modules to preserve the sign of correlations among expression proﬁles was created. Before the start of network analysis, the data samples have been assessed for the presence of outliers; those samples showing the dissimilarity in the group were discarded. The pairwise similarities of all DEGs were quantified by means of Pearson Correlation Coefficient (*r*) on the basis of expression profiles across all cancer samples; this resulting correlation matrix was transformed into an adjacency matrix with the following equation:

$$a_{\left( ij \right)}={|0.5+0.5 \times cor(x_{\left( i \right)}{,x}_{\left( j \right)})|}^{\beta}$$

where $a_{\left( ij \right)}$ is the adjacency value of gene *i* and gene j, and *β* is the weight that serves to highlight the strongest correlations.

The coexpression similarity was raised to a power of β, also called soft threshold power—which preserves the continuous nature of the coexpression relationships—to maintain high similarity measures as high adjacencies (lower similarity measures are pushed toward an adjacency of zero). To find the optimal β in the pickSoftThreshold function of WGCNA; β was tested in power interval between 1 and 20 and we finally selected power that satisfied the fit of scale free topology (that also maintained a high mean connectivity). Topological Overlap Matrix (TO)—a measure of network interconnectedness—was used to transform adjacency matrix into a coexpression distance matrix. The topological overlap based dissimilarity (1-TO) was then used as an input for the average linkage hierarchical clustering. Finally, modules were selected from branches of the resulting clustering tree. Because dynamic tree-cutting algorithm leads to more robust module assignments; therefore, to cut the branches of the hierarchical clustering tree, cutreeDynamic function was used that defined the modules as branches. The modules with very similar expression profiles were then merged with mergeCloseModules function as a standard procedure of WGCNA. A summary profile, or eigengene, was calculated by performing principal component analysis for each module. The resulting first principal component of the normalized gene expression matrix was retained as the representative of the module eigengene (ME). So, ME considered here is a weighted average of module gene expression profiles. All other parameters have been used with the default values in WGCNA. To adjudge the scale-free nature of degree distributions of the network, discrete power-law hypothesis was tested using poweRlaw software package [2].

**1. 2. Preservation of module analysis and their association with phenotypic trait**

The robustness and high quality of resulting coexpression modules were assessed and verified by means of modulePreservation function with 100 permutations. To ensure that the modules generated are not by chance, their reproducibility was examined by a resampling procedure [3] Two methods to generate Z-summary scores were used. First, module statistics of the merged dataset (reference dataset) was compared to the randomly generated modules in a test dataset, which comprised 100 random samples from the reference dataset. Second, we also replicated module preservation analysis over individual GEO datasets by assuming that they were test datasets. Further, to incorporate the OSCC phenotype status into the coexpression network and identify stage-associated modules, we tried to find correlation of each module with a disease phenotype by means of Pearson rank correlation. In general, stages I and II are described as early stage cancers, while stages III and IV as late stages. A binary indicator variable (early stage=0; late stage=1) was assigned for arrays and checked for any MEs if they were significantly correlated with that indicator. Modules that significantly correlate with a stage phenotype were labeled “candidate modules”. In our analysis, only one module (pink) was found to correlate with a stage and therefore was analyzed further. The gene coexpression network for the pink candidate module was then visualized by importing network data into Cytoscape v3.0.1 [4] using exportNetworkToCytoscape function.

**References**

1. Langfelder P, Horvath S: **WGCNA: an R package for weighted correlation network analysis**. *BMC Bioinformatics* 2008, **9**:559.

2. Gillespie CS: **Fitting heavy tailed distributions: the poweRlaw package**. 2014.

3. Langfelder P, Mischel PS, Horvath S: **When is hub gene selection better than standard meta-analysis?**. *PLoS One* 2013, **8**:e61505.

4. Shannon P, Markiel A, Ozier O, Baliga NS, Wang JT, Ramage D, Amin N, Schwikowski B, Ideker T: **Cytoscape: a software environment for integrated models of biomolecular interaction networks**. *Genome Res.* 2003, **13**:2498–504.
