# Supplementary material for: Integrated network analysis and logistic regression modeling identify stage-specific genes in Oral Squamous Cell Carcinoma
Source: BMC Med Genomics. 2015 Jul 16;8:39. doi: 10.1186/s12920-015-0114-0 (PMC4502639; doi:10.1186/s12920-015-0114-0)

Additional File 2 (Supplementary Figures)

Figure S1. Expression intensity distributions of arrays. Density plots of arrays of probe-level data before (A) and after (B) normalization.

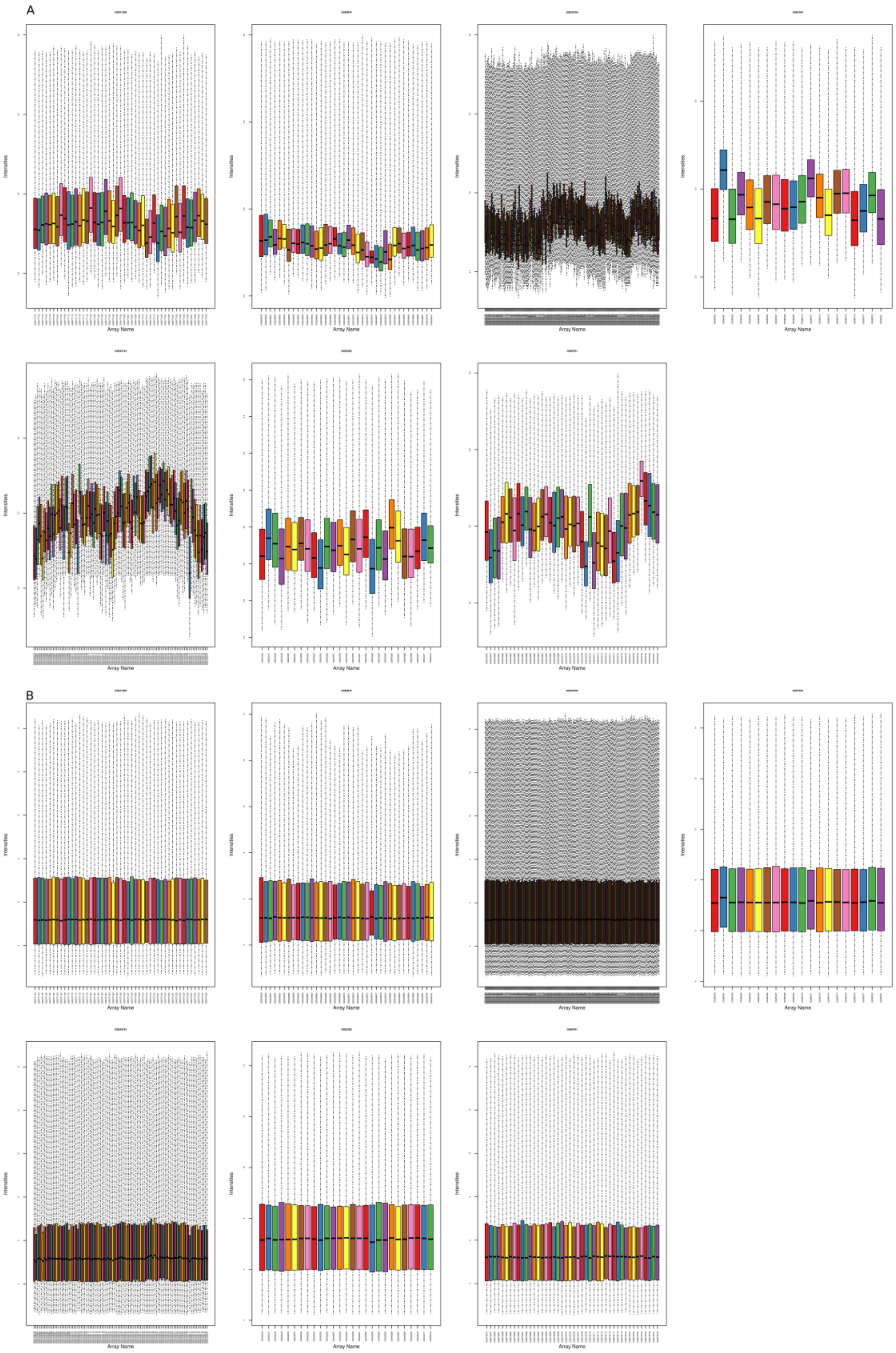

Figure S2.Expression intensity distributions of arrays. Box plots of arrays of probe-level data before (A) and after (B) normalization.

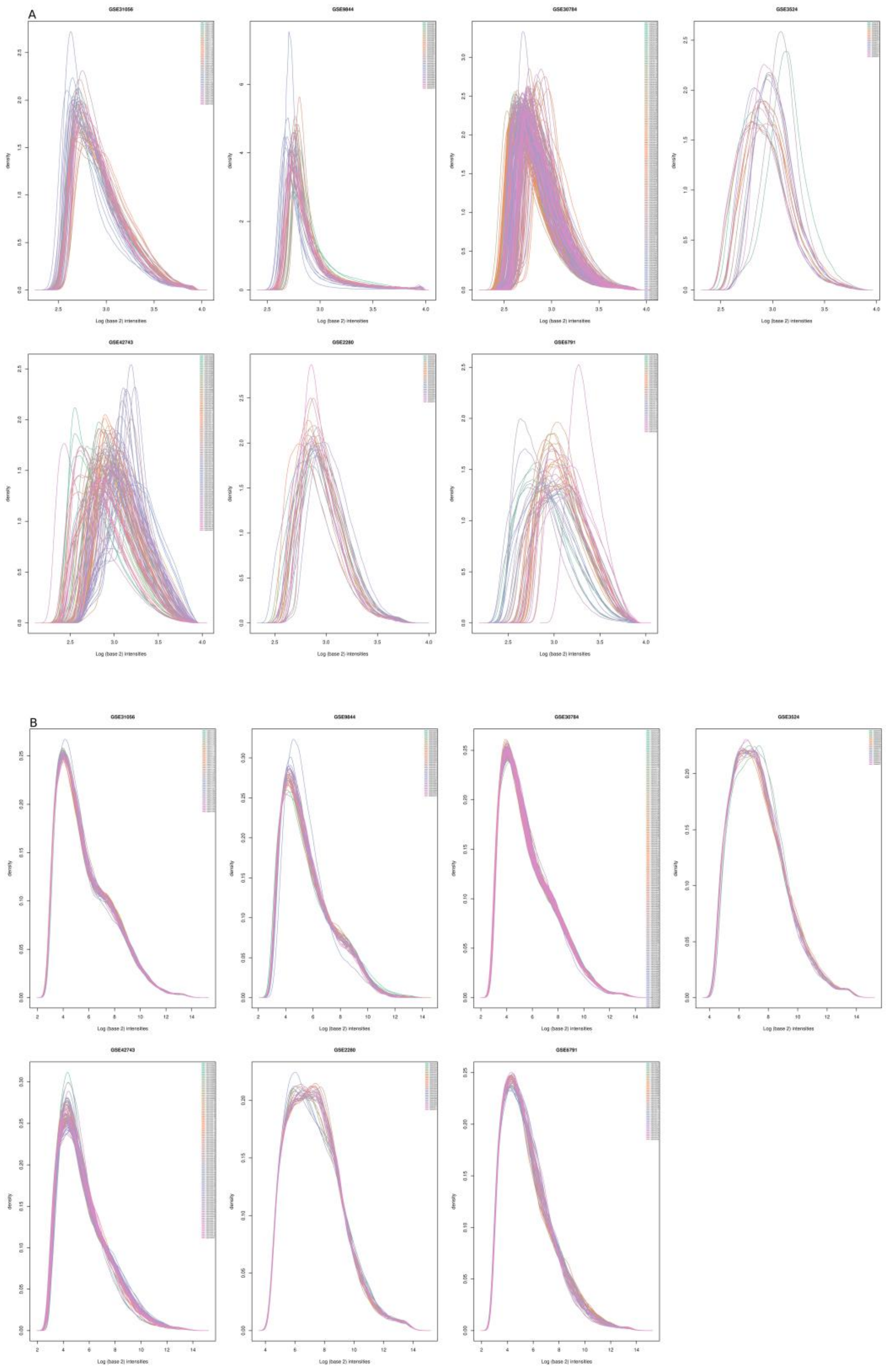

Figure S3. Heatmap plot analysis of merged gene expression data without (A) and with (B) batch effect removal approach. The hierarchical clustering was performed by means of gplots software package. Ward's method with Euclidean distance metric was used for the clustering. The samples are displayed on the X-axis while genes are listed on the Y-axis. The vertical branches of cluster, representing samples, are color coded according to group with “dark-red” and “forest-green” for cancer and normal samples, respectively. The resulting heatmap shows that the samples are clustered together by data source when they are simply merged (A); however, after the application of COMBAT, the influence of data source on grouping is significantly reduced (B). This COMBAT-merged method resulted in clustering of the samples into their distinct normal and cancer groups.

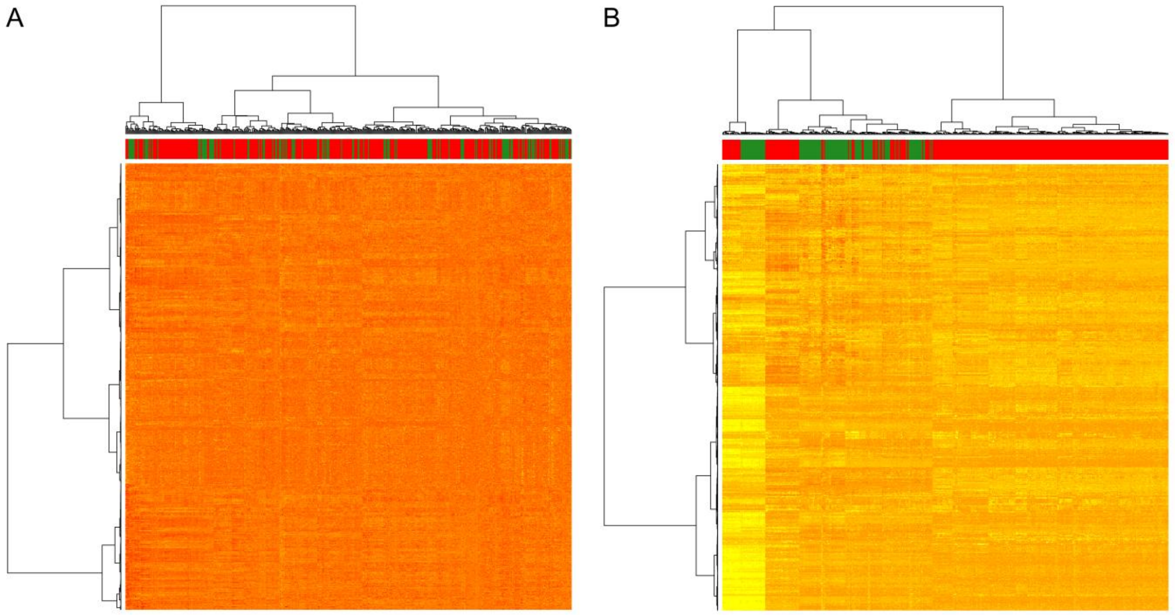

Figure S4. Relative log expression (RLE) boxplots. A plot indicating mean of RLE is not centred around zero and represent an uneven spread when they are simply merged (A); however, after the application of COMBAT, the mean of RLE plot was distributed around zero for all genes (B). This result is an indicative of removal of batch effect by COMBAT-merged method. Additionally, the plot highlighted the existence of 7 clear batches in simply combined datasets (A); however, COMBAT-implementation method greatly improved the appearance of plot (B). All samples are color coded based on biological variable of interest (healthy (red); cancer (green)).

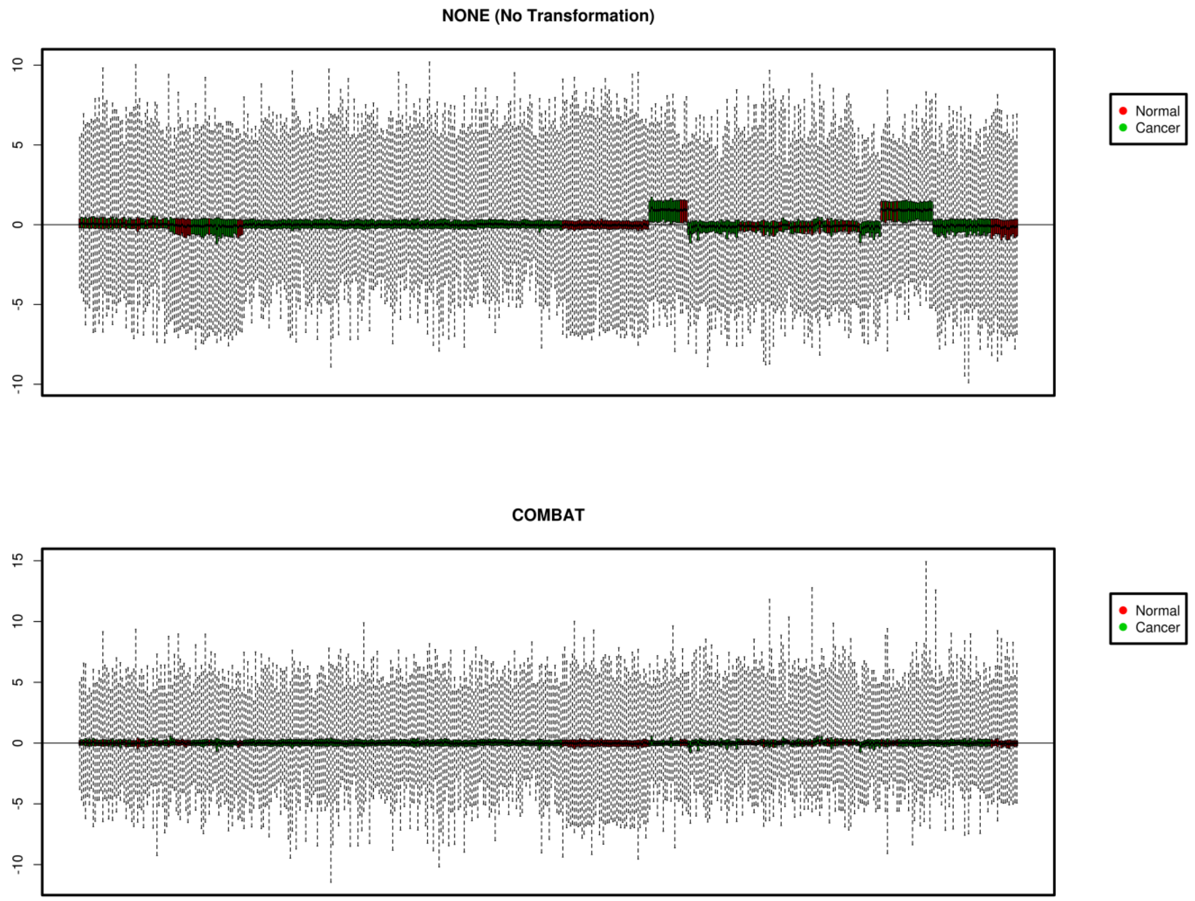

Figure S5. Volcano plot of differentially expressed genes (DEGs). The log2 fold change is plotted on the X-axis and the negative log10 adjusted p-value is plotted on the Y-axis. Cyan circles graphically display 1652 genes that satisfy both criteria of p-value (False Discovery Rate [FDR]<0.05) and fold change (>two-fold change).

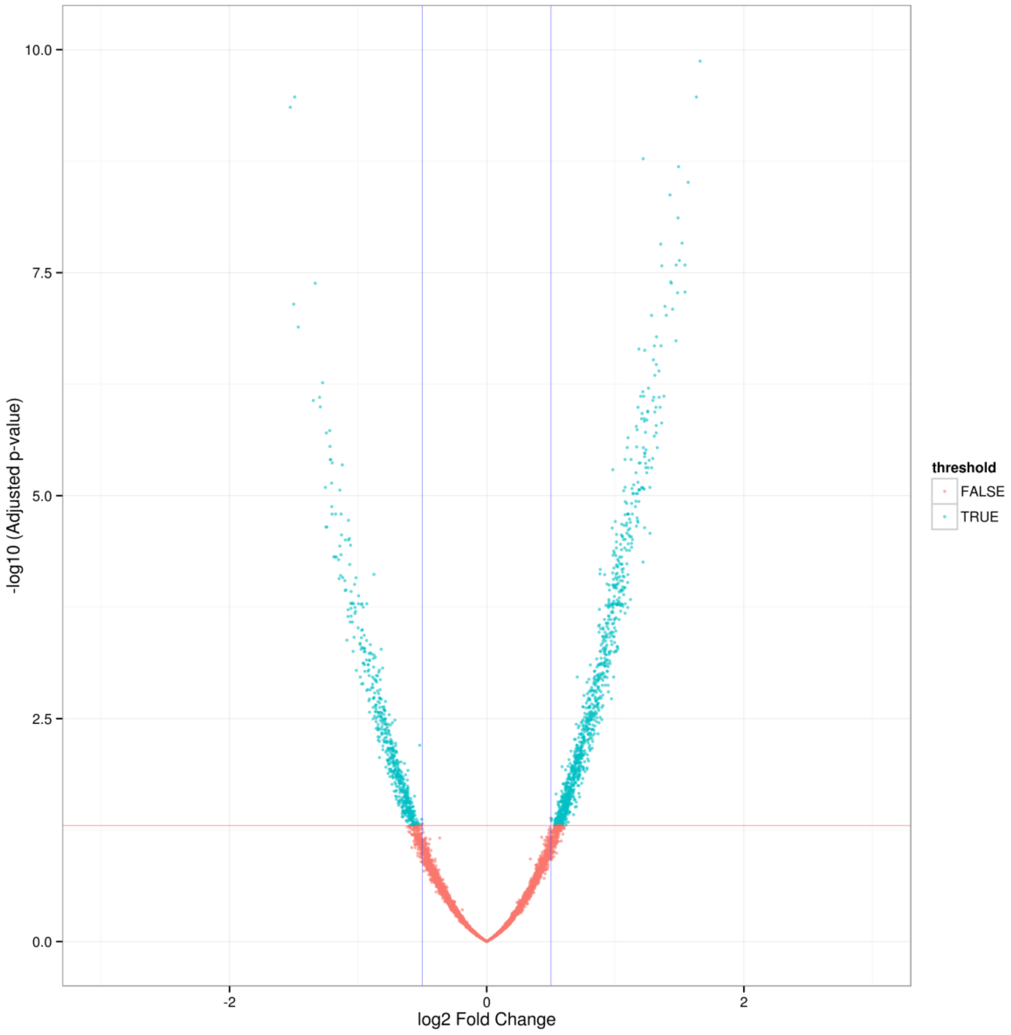

Figure S6. Clustering analysis of differentially expressed genes (DEGs). The hierarchical clustering was in the gplots software package. Ward's method with Euclidean distance metric was used for the clustering. The clustering of DEGs shows a distinct separation between normal and cancer groups. The samples are displayed on the Y-axis and genes are listed on X-axis. The vertical branches of cluster, representing samples, are color coded according to group with “dark-red” and “forest-green” for cancer and normal samples, respectively.

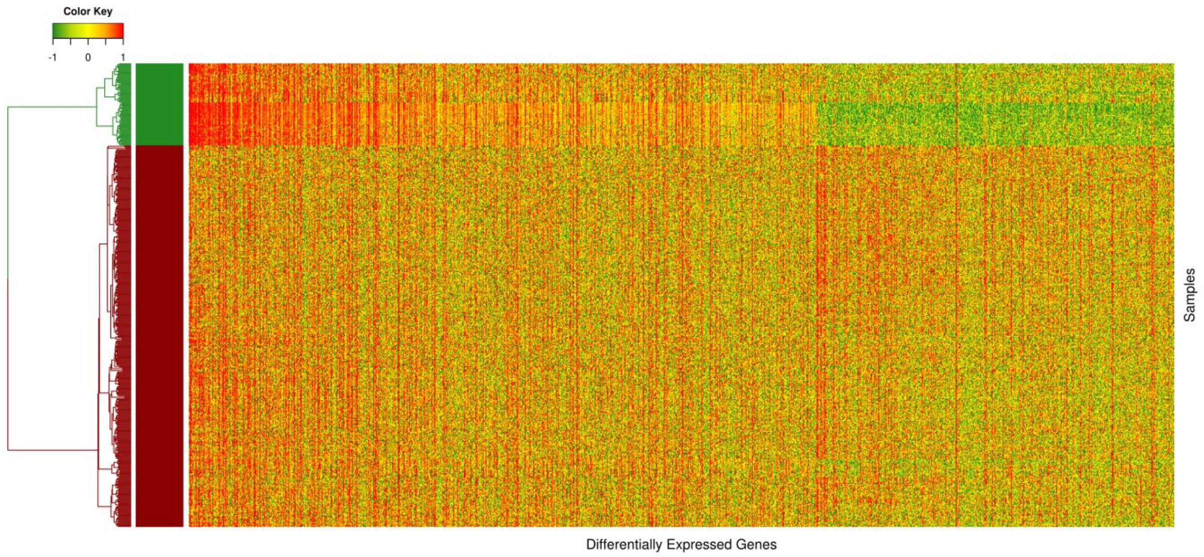

Figure S7. The node degree distribution of Oral Squamous Cell Carcinoma disease network. The number of nodes (i.e., genes) is plotted as a function of their degree which reflects a power-law like distribution; this is an indicative of scale-free network topology. The red line corresponds to a power-law distribution with parameters x_{min} = 44 and α = 6.62, where x_{min} is lower cut-off and α is the scaling parameter.

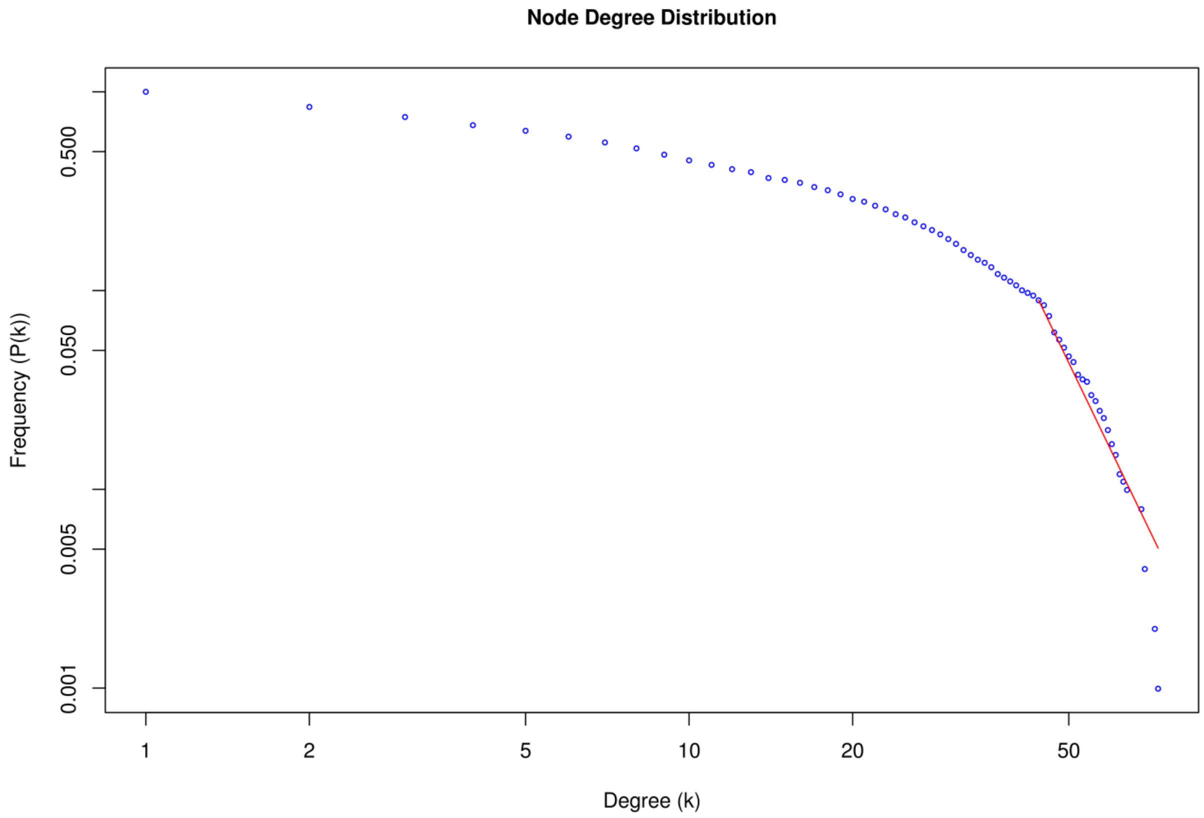

Figure S8. The expression heatmap plots of modules correlated with a stage. The rows correspond to genes and the columns to random samples. Here, in this figure, genes color coded green are underexpressed, while red indicates overexpression.

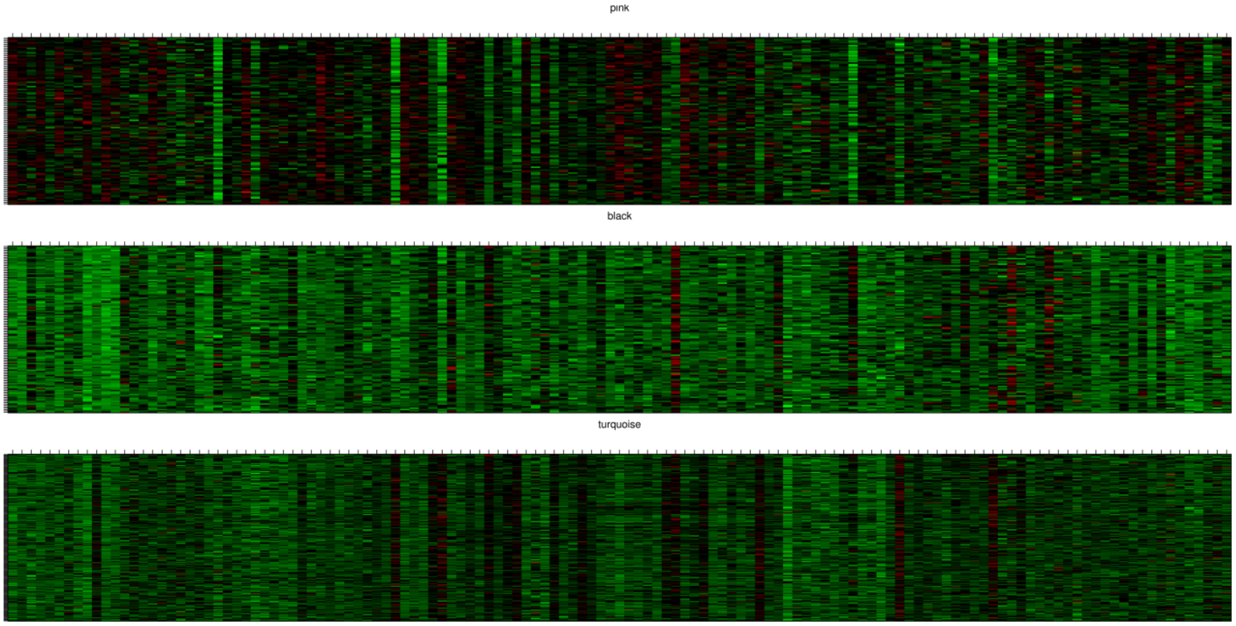

Figure S9. The enriched gene ontology (GO) terms obtained for hub genes. The enrichment analysis (Fisher exact test $p<0.05$) is carried out by means of GoStats software package and summarized using REVIGO.

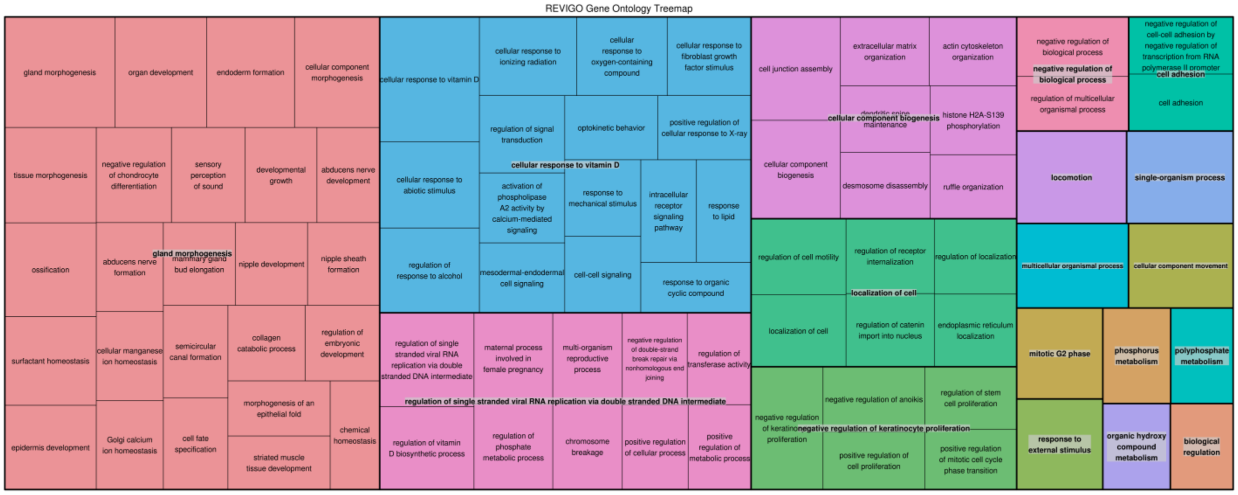

Supplement: Additional file 2: Figure S1. — (Supplementary Figures). Expression intensity distributions of arrays. Density plots of arrays of probe-level data before (A) and after (B) normalization. Figure S2.Expression intensity distributions of arrays. Box plots of arrays of probe-level data before (A) and after (B) normalization.Figure S3. Heatmap plot analysis of merged gene expression data without (A) and with (B) batch effect removal approach. The hierarchical clustering was performed by means of gplots software package. Ward's method with Euclidean distance metric was used for the clustering. The samples are displayed on the X-axis while genes are listed on the Y-axis. The vertical branches of cluster, representing samples, are color coded according to group with “dark-red” and “forest-green” for cancer and normal samples, respectively. The resulting heatmap shows that the samples are clustered together by data source when they are simply merged (A); however, after the application of COMBAT, the influence of data source on grouping is significantly reduced (B). This COMBAT-merged method resulted in clustering of the samples into their distinct normal and cancer groups. Figure S4. Relative log expression (RLE) boxplots. A plot indicating mean of RLE is not centred around zero and represent an uneven spread when they are simply merged (A); however, after the application of COMBAT, the mean of RLE plot was distributed around zero for all genes (B). This result is an indicative of removal of batch effect by COMBAT-merged method. Additionally, the plot highlighted the existence of 7 clear batches in simply combined datasets (A); however, COMBAT-implementation method greatly improved the appearance of plot (B). All samples are color coded based on biological variable of interest (healthy (red); cancer (green)). Figure S5. Volcano plot of differentially expressed genes (DEGs). The log2 fold change is plotted on the X-axis and the negative log10 adjusted p-value is plotted on the Y-axis. Cyan circles graphical [file 12920_2015_114_MOESM2_ESM.pdf]
