# Supplementary material for: Integrated network analysis and logistic regression modeling identify stage-specific genes in Oral Squamous Cell Carcinoma
Source: BMC Med Genomics. 2015 Jul 16;8:39. doi: 10.1186/s12920-015-0114-0 (PMC4502639; doi:10.1186/s12920-015-0114-0)
Supplement: Additional file 3: Table S1. — (Supplementary Tables). The Z-summary statistics of modules obtained against bootstrapped networks from the individual dataset from Gene Expression Omnibus (GEO). Table S2. The roles of hub genes (corresponding to pink module) highlighting their significance in OSCC and other cancers. Table S3. The perturbed pathways obtained in hub genes on the basis of combined Signaling Pathway Impact Analysis (SPIA) criteria after a False Discovery Rate correction of 1 % on the global p-values (pG). (pSize: number of genes in the pathway; NDE: number of DE genes in the pathway; pNDE: Probability to observe at least NDE genes in the pathway using a hypergeometric model; tA: observed total perturbation accumulation in the pathway; pPERT: probability to observe a total accumulation more extreme than tA by chance; pG: p-value obtained by combining pNDE and pPERT; pGFdr: False Discovery Rate; Status: Direction in which the pathway is perturbed). Table S4. The REVIGO summarized biological process (BP) categories terms for hub genes (corresponding to pink module). [file 12920_2015_114_MOESM3_ESM.pdf]

## Additional File3 (Supplementary Tables)

Table S1. The Z-summary statistics of modules obtained against bootstrapped networks from the individual dataset from Gene Expression Omnibus (GEO).

| Module Color | Module Size | GSE31056 | GSE9844 | GSE30784 | GSE3524 | GSE42743 | GSE2280 | GSE6791 |
|---|---|---|---|---|---|---|---|---|
| yellow | 145 | 33.89 | 11.66 | 44.04 | 12.94 | 17.63 | 20.61 | 19.15 |
| red | 130 | 23.02 | 9.48 | 25.92 | 18.60 | 16.25 | 19.22 | 13.25 |
| turquoise | 212 | 19.76 | 6.50 | 12.14 | 3.15 | 22.54 | 20.37 | 0.72 |
| purple | 85 | 19.10 | 7.81 | 14.59 | 11.86 | 13.36 | 17.09 | 5.74 |
| Black | 123 | 17.03 | 10.14 | 24.66 | 8.49 | 13.78 | 13.21 | 11.25 |
| magenta | 110 | 14.33 | 10.15 | 16.20 | 13.36 | 15.37 | 13.96 | 8.26 |
| Greenyellow | 70 | 12.20 | 11.66 | 24.06 | 14.80 | 13.78 | 14.95 | 10.96 |
| pink | 114 | 12.19 | 6.15 | 17.07 | 4.42 | 18.57 | 9.97 | 10.05 |
| blue | 339 | 10.87 | 12.54 | 12.46 | 4.10 | 25.21 | 9.78 | 20.82 |
| brown | 150 | 10.10 | 4.14 | 16.57 | 6.17 | 15.53 | 8.70 | 9.95 |
| grey | 98 | 7.53 | 1.86 | 4.09 | 2.93 | 8.69 | 2.68 | 2.70 |
| tan | 41 | 7.39 | 5.51 | 8.39 | 6.36 | 6.10 | 6.96 | 5.51 |
| gold | 100 | 5.28 | 1.93 | 4.63 | 3.32 | 8.49 | 2.03 | 6.72 |
| salmon | 35 | 4.87 | 5.70 | 8.31 | 5.78 | 9.61 | 7.20 | 4.50 |

Table S2. The roles of hub genes (corresponding to pink module) highlighting their significance in OSCC and other cancers.

| Hub Gene | Known Associated Disease | Reference |
|---|---|---|
| *MMP12* | OSCC/HNSCC | [1] |
| *MYO1B* | OSCC/HNSCC | [2] |
| *TNFRSF10B* | OSCC/HNSCC | [3] |
| *MET* | OSCC/HNSCC | [4] |
| *SNAI2* | OSCC/HNSCC | [5] |
| *LAMA3* | OSCC/HNSCC | [6] [7] |
| *CYP27B1* | OSCC/HNSCC | [8] |
| *IGF2BP3* | OSCC/HNSCC | [9] |
| *EGFR* | OSCC/HNSCC | [10] [11] |
| *CXCL5* | OSCC/HNSCC | [12] |
| *ITGB1* | OSCC/HNSCC | [13] |
| *IGF2BP2* | OSCC/HNSCC | [14] |
| *DUSP6* | OSCC | [15] |
| *PTHLH* | OSCC | [16] |
| *LAMC2* | OSCC | [16] |
| *MMP10* | OSCC | [16] |
| *IL8* | OSCC | [16] |
| *STK3* | OSCC | [17] |
| *HMGA2* | OSCC | [18] |
| *GNA12* | OSCC | [19] [20] |
| *GNB5* | OSCC | [21] |
| *RSF1* | OSCC | [22] |
| *HOXA1* | OSCC | [23] |
| *MYO5A* | OSCC | [24] |
| *SLC20A1* | OSCC | [25] |
| *IRS1* | OSCC | [26] |
| *DKK1* | OSCC | [27] |
| *CBX3* | HNSCC | [28] |
| *ABCC4* | HNSCC | [29] |
| *SEMA3C* | OSCC | [30] |
| *IL8* | OSCC | [31] |
| *SDC4* | breast tumors | [32] |

| | | |
|---|---|---|
| *DFNA5* | gastric tumors | [33] |
| *STC1* | colorectal tumors | [34] |
| *PTPRK* | colorectal tumors | [35] |
| *E2F3* | bladder tumors | [36] |
| *PLAG1* | salivary glands tumor | [37] |

Table S3. The perturbed pathways obtained in hub genes on the basis of combined Signaling Pathway Impact Analysis (SPIA) criteria after a False Discovery Rate correction of 1 % on the global p-values (pG). (pSize: number of genes in the pathway; NDE: number of DE genes in the pathway; pNDE: Probability to observe at least NDE genes in the pathway using a hypergeometric model; tA: observed total perturbation accumulation in the pathway; pPERT: probability to observe a total accumulation more extreme than tA by chance; pG: p-value obtained by combining pNDE and pPERT; pGFdr: False Discovery Rate; Status: Direction in which the pathway is perturbed).

| **Pathway Name** | **pSize** | **NDE** | **pNDE** | **tA** | **pPERT** | **pG** | **pGFdr** | **Status** | **Source Database** |
|---|---|---|---|---|---|---|---|---|---|
| mcalpain and friends in cell motility | 41 | 4 | $5.1\times10^{-05}$ | -11.14 | $3.0\times10^{-02}$ | $2.2\times10^{-05}$ | 0.00295 | Inhibited | Biocarta |
| a6b1 and a6b4 Integrin signaling | 32 | 4 | $1.8\times10^{-05}$ | -4.88 | $1.5\times10^{-01}$ | $4.0\times10^{-05}$ | 0.00295 | Inhibited | NCI Nature curated pathway |
| Small cell lung cancer | 82 | 5 | $5.6\times10^{-05}$ | 5.66 | $6.0\times10^{-02}$ | $4.5\times10^{-05}$ | 0.002957 | Activated | KEGG |
| Direct p53 effectors | 113 | 5 | $2.5\times10^{-04}$ | -3.73 | $1.7\times10^{-02}$ | $5.8\times10^{-05}$ | 0.00295 | Inhibited | NCI Nature curated pathway |
| Pathways in cancer | 294 | 8 | $1.0\times10^{-04}$ | 7.44 | $1.6\times10^{-01}$ | $2.0\times10^{-04}$ | 0.00836 | Activated | KEGG |

Table S4. The REVIGO summarized biological process (BP) categories terms for hub genes (corresponding to pink module).

| Revigo Representative GO Term | Child GO BP ID | Child GO BP Term Description | Hypergeometric P Value | No. Genes in GO Category |
|---|---|---|---|---|
| mitotic G2 phase | GO:0000085 | mitotic G2 phase | $1.16\times10^{-03}$ | 2 |
| cell adhesion | GO:0007155 | cell adhesion | $5.85\times10^{-03}$ | 11 |
| | GO:1900387 | negative regulation of cell-cell adhesion by negative regulation of transcription from RNA polymerase II promoter | $5.79\times10^{-03}$ | 1 |
| multicellular organismal process | GO:0032501 | multicellular organismal process | $2.85\times10^{-04}$ | 41 |
| locomotion | GO:0040011 | Locomotion | $1.55\times10^{-04}$ | 17 |
| cellular component biogenesis | GO:0044085 | cellular component biogenesis | $5.06\times10^{-04}$ | 19 |
| | GO:0035978 | histone H2A-S139 phosphorylation | $5.79\times10^{-03}$ | 1 |
| | GO:0097062 | dendritic spine maintenance | $5.79\times10^{-03}$ | 1 |
| | GO:0031529 | ruffle organization | $8.38\times10^{-03}$ | 2 |
| | GO:0034329 | cell junction assembly | $3.44\times10^{-04}$ | 6 |
| | GO:0030198 | extracellular matrix organization | $5.13\times10^{-03}$ | 5 |
| | GO:0030036 | actin cytoskeleton organization | $5.56\times10^{-03}$ | 7 |
| | GO:0035921 | desmosome disassembly | $5.79\times10^{-03}$ | 1 |

| | | | | |
|---|---|---|---|---|
| single-organism process | GO:0044699 | single-organism process | $2.04\times10^{-04}$ | 59 |
| localization of cell | GO:0051674 | localization of cell | $2.79\times10^{-03}$ | 12 |
| | GO:0051643 | endoplasmic reticulum localization | $5.79\times10^{-03}$ | 1 |
| | GO:2000145 | regulation of cell motility | $1.95\times10^{-03}$ | 8 |
| | GO:0032879 | regulation of localization | $4.77\times10^{-03}$ | 15 |
| | GO:0035412 | regulation of catenin import into nucleus | $3.75\times10^{-03}$ | 2 |
| | GO:0002090 | regulation of receptor internalization | $3.30\times10^{-03}$ | 2 |
| biological regulation | GO:0065007 | biological regulation | $9.42\times10^{-03}$ | 49 |
| cellular response to vitamin D | GO:0071305 | cellular response to vitamin D | $2.15\times10^{-05}$ | 3 |
| | GO:0003131 | mesodermal-endodermal cell signaling | $5.79\times10^{-03}$ | 1 |
| | GO:0043006 | activation of phospholipase A2 activity by calcium-mediated signalling | $5.79\times10^{-03}$ | 1 |
| | GO:0071214 | cellular response to abiotic stimulus | $6.01\times10^{-04}$ | 6 |
| | GO:0007267 | cell-cell signalling | $6.62\times10^{-03}$ | 12 |
| | GO:0030522 | intracellular receptor signaling pathway | $7.23\times10^{-03}$ | 5 |
| | GO:0007634 | optokinetic behaviour | $5.79\times10^{-03}$ | 1 |
| | GO:0009966 | regulation of signal transduction | $3.37\times10^{-03}$ | 18 |
| | GO:0033993 | response to lipid | $7.93\times10^{-03}$ | 8 |
| | GO:1901419 | regulation of response to alcohol | $6.80\times10^{-03}$ | 2 |

| | | | | |
|---|---|---|---|---|
| | GO:1901701 | cellular response to oxygen-containing compound | $2.14\times10^{-03}$ | 10 |
| | GO:0044344 | cellular response to fibroblast growth factor stimulus | $3.35\times10^{-03}$ | 5 |
| | GO:0071479 | cellular response to ionizing radiation | $1.35\times10^{-03}$ | 3 |
| | GO:2000685 | positive regulation of cellular response | $5.79\times10^{-03}$ | 1 |
| | GO:0009612 | response to mechanical stimulus | $6.54\times10^{-03}$ | 4 |
| | GO:0014070 | response to organic cyclic compound | $8.12\times10^{-03}$ | 8 |
| gland morphogenesis | GO:0022612 | gland morphogenesis | $2.72\times10^{-05}$ | 6 |
| | GO:0043129 | surfactant homeostasis | $9.09\times10^{-04}$ | 2 |
| | GO:0030574 | collagen catabolic process | $6.52\times10^{-03}$ | 3 |
| | GO:0007605 | sensory perception of sound | $2.36\times10^{-03}$ | 4 |
| | GO:0001708 | cell fate specification | $5.99\times10^{-03}$ | 3 |
| | GO:0001503 | Ossification | $6.13\times10^{-04}$ | 7 |
| | GO:0021599 | abducens nerve formation | $5.79\times10^{-03}$ | 1 |
| | GO:0048589 | developmental growth | $2.78\times10^{-03}$ | 6 |
| | GO:0032331 | negative regulation of chondrocyte differentiation | $2.10\times10^{-03}$ | 2 |
| | GO:0030026 | cellular manganese ion homeostasis | $5.79\times10^{-03}$ | 1 |
| | GO:0032468 | Golgi calcium ion homeostasis | $5.79\times10^{-03}$ | 1 |

| | | | | |
|---|---|---|---|---|
| | GO:0001706 | endoderm formation | $2.28\times10^{-04}$ | 3 |
| | GO:0060876 | semicircular canal formation | $5.79\times10^{-03}$ | 1 |
| | GO:0008544 | epidermis development | $1.30\times10^{-03}$ | 5 |
| | GO:0045995 | regulation of embryonic development | $7.68\times10^{-03}$ | 3 |
| | GO:0060649 | mammary gland bud elongation | $5.79\times10^{-03}$ | 1 |
| | GO:0060659 | nipple sheath formation | $5.79\times10^{-03}$ | 1 |
| | GO:0048878 | chemical homeostasis | $9.57\times10^{-03}$ | 9 |
| | GO:0060618 | nipple development | $5.79\times10^{-03}$ | 1 |
| | GO:0021560 | abducens nerve development | $5.79\times10^{-03}$ | 1 |
| | GO:0032989 | cellular component morphogenesis | $2.94\times10^{-04}$ | 14 |
| | GO:0060571 | morphogenesis of an epithelial fold | $7.71\times10^{-03}$ | 2 |
| | GO:0014706 | striated muscle tissue development | $8.84\times10^{-03}$ | 6 |
| | GO:0048513 | organ development | $1.59\times10^{-04}$ | 23 |
| | GO:0048729 | tissue morphogenesis | $5.42\times10^{-04}$ | 9 |
| negative regulation of keratinocyte proliferation | GO:0010839 | negative regulation of keratinocyte proliferation | $4.87\times10^{-04}$ | 2 |
| | GO:1901992 | positive regulation of mitotic cell cycle phase transition | $8.39\times10^{-03}$ | 2 |
| | GO:2000811 | negative regulation of anoikis | $3.30\times10^{-03}$ | 2 |
| | GO:0008284 | positive regulation of cell proliferation | $6.17\times10^{-03}$ | 9 |

| | | | | |
|---|---|---|---|---|
| | GO:0072091 | regulation of stem cell proliferation | $7.09\times10^{-03}$ | 3 |
| polyphosphate metabolism | GO:0006797 | polyphosphate metabolic process | $5.79\times10^{-03}$ | 1 |
| organic hydroxy compound metabolism | GO:1901615 | organic hydroxy compound metabolic process | $6.68\times10^{-03}$ | 7 |
| negative regulation of biological process | GO:0048519 | negative regulation of biological process | $3.17\times10^{-03}$ | 26 |
| | GO:0051239 | regulation of multicellular organismal process | $5.09\times10^{-03}$ | 18 |
| response to external stimulus | GO:0009605 | response to external stimulus | $1.18\times10^{-03}$ | 14 |
| cellular component movement | GO:0006928 | cellular component movement | $4.80\times10^{-04}$ | 17 |
| phosphorus metabolism | GO:0006793 | phosphorus metabolic process | $3.71\times10^{-03}$ | 21 |
| regulation of single stranded viral RNA replication via double stranded DNA intermediate | GO:0045091 | regulation of single stranded viral RNA replication via double stranded DNA intermediate | $4.87\times10^{-04}$ | 2 |
| | GO:0060556 | regulation of vitamin D biosynthetic process | $1.16\times10^{-03}$ | 2 |
| | GO:0019220 | regulation of phosphate metabolic process | $2.92\times10^{-03}$ | 14 |
| | GO:0048522 | positive regulation of cellular process | $7.99\times10^{-03}$ | 19 |
| | GO:0031052 | chromosome breakage | $5.79\times10^{-03}$ | 1 |
| | GO:0060135 | maternal process involved in female | $2.07\times10^{-03}$ | 3 |

| | | | | |
|---|---|---|---|---|
| | | pregnancy | | |
| | GO:0051338 | regulation of transferase activity | $6.31\times10^{-03}$ | 9 |
| | GO:0009893 | positive regulation of metabolic process | $9.94\times10^{-03}$ | 18 |
| | GO:2001033 | negative regulation of double-strand break repair via nonhomologous end joining | $5.79\times10^{-03}$ | 1 |
| | GO:0044703 | multi-organism reproductive process | $4.25\times10^{-03}$ | 5 |

## References


1. Impola U, Uitto VJ, Hietanen J, Hakkinen L, Zhang L, Larjava H, Isaka K, Saarialho-Kere U: **Differential expression of matrilysin-1 (MMP-7), 92 kD gelatinase (MMP-9), and metalloelastase (MMP-12) in oral verrucous and squamous cell cancer**. *J. Pathol.* 2004, **202**:14–22.

2. Ohmura G, Tsujikawa T, Yaguchi T, Kawamura N, Mikami S, Sugiyama J, Nakamura K, Kobayashi A, Iwata T, Nakano H, Shimada T, Hisa Y, Kawakami Y: **Aberrant myosin 1b expression promotes cell migration and lymph node metastasis of HNSCC**. *Mol. Cancer Res.* 2014, **189**:285–90.

3. Schabath MB, Giuliano AR, Thompson ZJ, Amankwah EK, Gray JE, Fenstermacher DA, Jonathan KA, Beg AA, Haura EB: **TNFRSF10B polymorphisms and haplotypes associated with increased risk of death in non-small cell lung cancer**. *Carcinogenesis* 2013, **34**:2525–30.

4. Morello S, Olivero M, Aimetti M, Bernardi M, Berrone S, Di Renzo MF, Giordano S: **MET receptor is overexpressed but not mutated in oral squamous cell carcinomas**. *J. Cell. Physiol.* 2001, **189**:285–90.

5. Wang C, Liu X, Huang H, Ma H, Cai W, Hou J, Huang L, Dai Y, Yu T, Zhou X: **Deregulation of Snai2 is associated with metastasis and poor prognosis in tongue squamous cell carcinoma**. *Int. J. Cancer* 2012, **130**:2249–58.

6. Li R, Ochs MF, Ahn SM, Hennessey P, Tan M, Soudry E, Gaykalova DA, Uemura M, Brait M, Shao C, Westra W, Bishop J, Fertig EJ, Califano JA: **Expression microarray analysis reveals alternative splicing of LAMA3 and DST genes in head and neck squamous cell carcinoma**. *PLoS One* 2014, **9**:e91263.

7. Moller-Levet CS, Betts GNJ, Harris AL, Homer JJ, West CML, Miller CJ: **Exon array analysis of head and neck cancers identifies a hypoxia related splice variant of LAMA3 associated with a poor prognosis**. *PLoS Comput. Biol.* 2009, **5**:e1000571.

8. Zeljic K, Supic G, Stamenkovic Radak M, Jovic N, Kozomara R, Magic Z: **Vitamin D receptor, CYP27B1 and CYP24A1 genes polymorphisms association with oral cancer risk and survival**. *J. Oral Pathol. Med.* 2012, **41**:779–87.

9. Lin C-Y, Chen S-T, Jeng Y-M, Yeh C-C, Chou H-Y, Deng Y-T, Chang C-C, Kuo MY-P: **Insulin-like growth factor II mRNA-binding protein 3 expression promotes tumor formation and invasion and predicts poor prognosis in oral squamous cell carcinoma**. *J. Oral Pathol. Med. Pathol. Med.* 2011, **40**:699–705.

10. Herbst RS: **Review of epidermal growth factor receptor biology**. *Int. J. Radiat. Oncol. Biol. Phys.* 2004, **59**:21–6.

11. Erman M: **Molecular mechanisms of signal transduction: epidermal growth factor receptor family, vascular endothelial growth factor family, Kit, platelet-derived growth factor receptor, Ras**. *J. BUON* 2007, **12 Suppl 1**:S83–94.

12. Miyazaki H, Patel V, Wang H, Edmunds RK, Gutkind JS, Yeudall WA: **Down-regulation of CXCL5 inhibits squamous carcinogenesis**. *Cancer Res.* 2006, **66**:4279–84.

13. Hunt S, Jones A V, Hinsley EE, Whawell SA, Lambert DW: **MicroRNA-124 suppresses oral squamous cell carcinoma motility by targeting ITGB1**. *FEBS Lett.* 2011, **585**:187–92.

14. Alajez NM, Shi W, Wong D, Lenarduzzi M, Waldron J, Weinreb I, Liu F-F: **Lin28b promotes head and neck cancer progression via modulation of the insulin-like growth factor survival pathway**. *Oncotarget* 2012, **3**:1641–52.

15. Baba T, Kawaguchi M, Fukushima T, Sato Y, Orikawa H, Yorita K, Tanaka H, Lin C-Y, Sakoda S, Kataoka H: **Loss of membrane-bound serine protease inhibitor HAI-1 induces oral squamous cell carcinoma cells' invasiveness**. *J. Pathol.* 2012, **228**:181–92.

16. Ye H, Yu T, Temam S, Ziober BL, Wang J, Schwartz JL, Mao L, Wong DT, Zhou X: **Transcriptomic dissection of tongue squamous cell carcinoma**. *BMC Genomics* 2008, **9**:69.

17. Lohavanichbutr P, Houck J, Doody DR, Wang P, Mendez E, Futran N, Upton MP, Holsinger FC, Schwartz SM, Chen C: **Gene expression in uninvolved oral mucosa of OSCC patients facilitates identification of markers predictive of OSCC outcomes**. *PLoS One* 2012, **7**:e46575.

18. Sterenczak KA, Eckardt A, Kampmann A, Willenbrock S, Eberle N, Länger F, Kleinschmidt S, Hewicker-Trautwein M, Kreipe H, Nolte I, Murua Escobar H, Gellrich NC: **HMGA1 and HMGA2 expression and comparative analyses of HMGA2, Lin28 and let-7 miRNAs in oral squamous cell carcinoma**. *BMC Cancer* 2014, **14**:694.

19. Cheong SC, Chandramouli GVR, Saleh A, Zain RB, Lau SH, Sivakumaren S, Pathmanathan R, Prime SS, Teo SH, Patel V, Gutkind JS: **Gene expression in human oral squamous cell carcinoma is influenced by risk factor exposure**. *Oral Oncol.* 2009, **45**:712–9.

20. Gan CP, Zain RB, Abraham MT, Patel V, Gutkind JS, Cheong SC, Chong CE, Hamid S, Teo SH: **Expression of GNA12 and its role in oral cancer**. *Oral Oncol.* 2011, **47**:S114–S115.

21. Yamano Y, Uzawa K, Shinozuka K, Fushimi K, Ishigami T, Nomura H, Ogawara K, Shiiba M, Yokoe H, Tanzawa H: **Hyaluronan-mediated motility: a target in oral squamous cell carcinoma**. *Int. J. Oncol.* 2008, **32**:1001–9.

22. Fang F-M, Li C-F, Huang H-Y, Lai M-T, Chen C-M, Chiu I-W, Wang T-L, Tsai F-J, Shih I-M, Sheu JJ-C: **Overexpression of a chromatin remodeling factor, RSF-1/HBXAP, correlates with aggressive oral squamous cell carcinoma**. *Am. J. Pathol.* 2011, **178**:2407–15.

23. Bitu CC, Destro MF de SS, Carrera M, da Silva SD, Graner E, Kowalski LP, Soares FA, Coletta RD: **HOXA1 is overexpressed in oral squamous cell carcinomas and its expression is correlated with poor prognosis**. *BMC Cancer* 2012, **12**:146.

24. Méndez E, Lohavanichbutr P, Fan W, Houck JR, Rue TC, Doody DR, Futran ND, Upton MP, Yueh B, Zhao LP, Schwartz SM, Chen C: **Can a metastatic gene expression profile outperform tumor size as a predictor of occult lymph node metastasis in oral cancer patients?**. *Clin. Cancer Res.* 2011, **17**:2466–73.

25. Jiang L, Dai Y, Liu X, Wang C, Wang A, Chen Z, Heidbreder CE, Kolokythas A, Zhou X: **Identification and experimental validation of G protein alpha inhibiting activity polypeptide 2 (GNAI2) as a microRNA-138 target in tongue squamous cell carcinoma**. *Hum. Genet.* 2011, **129**:189–97.

26. Chang K-W, Chu T-H, Gong N-R, Chiang W-F, Yang C-C, Liu C-J, Wu C-H, Lin S-C: **miR-370 modulates insulin receptor substrate-1 expression and inhibits the tumor phenotypes of oral carcinoma**. *Oral Dis.* 2013, **19**:611–9.

27. Ogoshi K, Kasamatsu A, Iyoda M, Sakuma K, Yamatoji M, Sakamoto Y, Ogawara K, Shiiba M, Tanzawa H, Uzawa K: **Dickkopf-1 in human oral cancer**. *Int. J. Oncol.* 2011, **39**:329–336.

28. Shimizu S, Seki N, Sugimoto T, Horiguchi S, Tanzawa H, Hanazawa T, Okamoto Y: **Identification of molecular targets in head and neck squamous cell carcinomas based on genome-wide gene expression profiling**. *Oncol. Rep.* 2007, **18**:1489–97.

29. Sethi S, Benninger MS, Lu M, Havard S, Worsham MJ: **Noninvasive molecular detection of head and neck squamous cell carcinoma: an exploratory analysis**. *Diagnostic Mol. Pathol.* 2009, **18**:81–7.

30. Roberg K, Ceder R, Farnebo L, Norberg-Spaak L, Grafström RC: **Multiple genotypic aberrances associate to terminal differentiation-deficiency of an oral squamous cell carcinoma in serum-free culture**. *Differentiation* 2008, **76**:868–80.

31. St John MAR, Li Y, Zhou X, Denny P, Ho C-M, Montemagno C, Shi W, Qi F, Wu B, Sinha U, Jordan R, Wolinsky L, Park N-H, Liu H, Abemayor E, Wong DTW: **Interleukin 6 and interleukin 8 as potential biomarkers for oral cavity and oropharyngeal squamous cell carcinoma**. *Arch. Otolaryngol. Head. Neck Surg.* 2004, **130**:929–35.

32. Da Cunha JPC, Galante PAF, De Souza JES, Pieprzyk M, Carraro DM, Old LJ, Camargo AA, De Souza SJ: **The human cell surfaceome of breast tumors**. *Biomed Res. Int.* 2013, **2013**.

33. Akino K, Toyota M, Suzuki H, Imai T, Maruyama R, Kusano M, Nishikawa N, Watanabe Y, Sasaki Y, Abe T, Yamamoto E, Tarasawa I, Sonoda T, Mori M, Imai K, Shinomura Y, Tokino T: **Identification of DFNA5 as a target of epigenetic inactivation in gastric cancer**. *Cancer Sci.* 2007, **98**:88–95.

34. Peña C, Céspedes MV, Lindh MB, Kiflemariam S, Mezheyeuski A, Edqvist P-H, Hägglöf C, Birgisson H, Bojmar L, Jirström K, Sandström P, Olsson E, Veerla S, Gallardo A, Sjöblom T, Chang AC-M, Reddel RR, Mangues R, Augsten M, Ostman A: **STC1 expression by cancer-associated fibroblasts drives metastasis of colorectal cancer**. *Cancer Res.* 2013, **73**:1287–97.

35. Flavell JR, Baumforth KRN, Wood VHJ, Davies GL, Wei W, Reynolds GM, Morgan S, Boyce A, Kelly GL, Young LS, Murray PG: **Down-regulation of the TGF-beta target gene, PTPRK, by the Epstein-Barr virus encoded EBNA1 contributes to the growth and survival of Hodgkin lymphoma cells**. *Blood* 2008, **111**:292–301.

36. Huang L, Luo J, Cai Q, Pan Q, Zeng H, Guo Z, Dong W, Huang J, Lin T: **MicroRNA-125b suppresses the development of bladder cancer by targeting E2F3**. *Int. J. Cancer* 2011, **128**:1758–69.

37. Voz ML, Mathys J, Hensen K, Pendeville H, Van Valckenborgh I, Van Huffel C, Chavez M, Van Damme B, De Moor B, Moreau Y, Van de Ven WJM: **Microarray screening for target genes of the proto-oncogene PLAG1**. *Oncogene* 2004, **23**:179–91.
